# Synthesis of New C-3 Substituted Kynurenic Acid Derivatives

**DOI:** 10.3390/molecules25040937

**Published:** 2020-02-19

**Authors:** Bálint Lőrinczi, Antal Csámpai, Ferenc Fülöp, István Szatmári

**Affiliations:** 1 Institute of Pharmaceutical Chemistry and Research Group for Stereochemistry, Hungarian Academy of Sciences, University of Szeged, Eötvös u. 6, H-6720 Szeged, Hungary; lorinczi.balint@pharm.u-szeged.hu (B.L.); fulop@pharm.u-szeged.hu (F.F.); 2 Institute of Pharmaceutical Chemistry, University of Szeged, Interdisciplinary Excellence Center, Eötvös u. 6, H-6720 Szeged, Hungary; 3 Department of Inorganic Chemistry, Eötvös Loránd University (ELTE), Pázmány P. sétány 1/A, H-1117 Budapest, Hungary; csampai@caesar.elte.hu

**Keywords:** kynurenic acid, Mannich reaction, neuroprotective activity, DFT calculations

## Abstract

The application of kynurenic acid (KYNA) as an electron-rich aromatic system in the modified Mannich reaction has been examined. The extension possibility of the reaction was tested by using amines occurring in a number of bioactive products, such as morpholine, piperidine, or *N*-methylpiperazine and aldehydes of markedly different reactivities, like formaldehyde and benzaldehyde. The influence of substituents attached to position 3 on the aminoalkylation was also investigated. Thus, reactions of 3-carbamoyl-substituted precursors with tertiary amine containing side-chains were also tested to afford new KYNA derivatives with two potential cationic centers. By means of NMR spectroscopic measurements, supported by DFT calculations, the dominant tautomer form of KYNA derivatives was also determined.

## 1. Introduction

KYNA (kynurenic acid) is an endogenous product of the tryptophan (TRP) metabolism, a pathway known to be responsible for the production of nicotinamide adenine dinucleotide (NAD^+^) and NAD phosphate (NADP^+^) [1,2]. In this pathway, TRP is converted into various compounds, including L-kynurenine (L-KYN), which can be metabolised in two separate ways. One furnishes KYNA, whereas the other gives 3-hydroxykynurenine (3-OH-KYN) and quinolinic acid (QUIN), the precursors of NAD [3,4].

Among the important features of KYNA, one is that it is one of the few known endogenous excitatory amino acid receptor blockers with a broad spectrum of antagonistic properties in supraphysiological concentrations. One of its confirmed sites of action is the α-7-nicotinic acetylcholine (α-7-nACh) receptor and, interestingly, the other, identified recently, is a higher-affinity positive modulatory binding site at the α-amino-3-hydroxy-5-methyl-4-isoxazolepropionic acid (AMPA) receptor [5].

Since KYNA is a neuroprotective agent able to prevent neuronal loss following excitotoxic, ischemia-induced, and infectious neuronal injuries [6,7], there has recently been increasing interest in the synthesis and pharmacological studies of KYNA derivatives. The substitution of KYNA at positions 5–8 were achieved by starting from the corresponding aniline via the modified Conrad–Limpach method [8,9,10]. The hydroxy group at position 4 was transformed to ether [10,11,12] or amine functions [13,14,15], while the carboxylic function at position 2 was mostly modified into the corresponding esters [10,11,12] or amides [16,17,18,19,20,21].

Formally, KYNA can be considered to be a nitrogen-containing 1-naphthol derivative. In our previous studies, 1-naphthol and its *N*-containing analogues were successfully applied in the modified Mannich reaction (*m*Mr) [22] leading to the corresponding aminonaphthols [23], aminoquinolinols or aminoisoquinolinols [24,25]. A similar transformation starting from xanthurenic acid has been described by Schmitt et al. [26]. They managed to perform regioselective aminoalkylation at position 3 on this substrate, by using benzyl-protection of 8-hydroxyl group. Our present aim was to examine the possibility of aminoalkylation of KYNA derivatives at position 3 via a *m*Mr approach. Since it is postulated that the reactivity of KYNA derivatives is influenced by tautomerism, we also envisaged identifying the tautomeric form dominant in solution. Finally, the reactivity of representative KYNA amides, carrying *N*-dialkylaminoalkyl-type side-chain possibly protonated under the standard reaction conditions, was also planned to be tested as these substrates with slightly decreased electron density at C-3 might display a decreased reactivity compared to that produced by a KYNA ester.

## 2. Results

In our first experiments, ethyl 4-oxo-1,4-dihydroquinoline-2-carboxylate (**1**) was reacted with 2-morpholinoethylamine in the presence of aqueous formaldehyde (22% solution). The reaction was conducted in different solvents (acetonitrile, *N*,*N*-dimethylformamide, EtOH, and 1,4-dioxane) at different temperatures (60 °C, 80 °C, and 110 °C). The optimised reaction conditions were found to be reflux temperature, using 1,4-dioxane as solvent, and 5 h reaction time. All reaction conditions led to the formation of 3-(((2-morpholinoethyl)amino)methyl)-4-oxo-1,4-dihydroquinoline-2-carboxylic acid (**2**) as the single product. It is interesting to note that the media was basic enough to lead to the hydrolysis of the ester function.

**Scheme 1:**
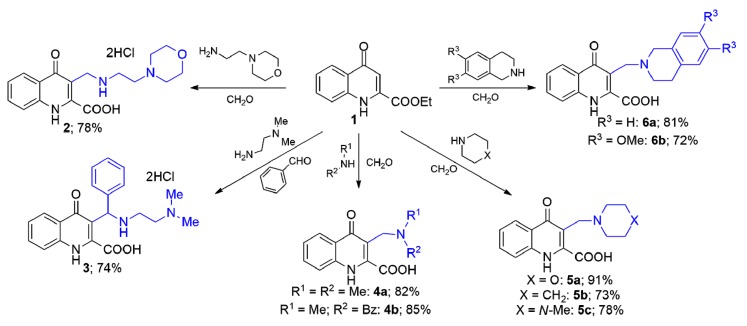
Syntheses of kynurenic acid (KYNA) Mannich derivatives.

To explore the scope and limitation of the transformation, **1** was first reacted with *N*,*N*-dimethylethane-1,2-diamine in the presence of benzaldehyde. The desired amino acid derivative **3** was isolated in a yield of 74%. Next, starting from dimethylamine or *N*-benzylmethylamine in the *m*Mr, the insertion of cationic centers in one-carbon distance at position 3 can be achieved. The structures of the formed amino acids **4a** and **4b** are depicted in Scheme 1.

The extension possibility of the reaction was further tested by starting from cyclic secondary amines such as morpholine, piperidine or *N*-methylpiperazine leading to **5a**, **5b** or **5c**, respectively. As last representative amines 1,2,3,4-tetrahydroisoquinoline and its dimethoxy analogue were chosen as aromatic fused cyclic secondary amines. In these cases, relatively long reaction times (8 h and 10 h) led to the formation of 3-((3,4-dihydroisoquinolin-2(1H)-yl)methyl)-4-oxo-1,4-dihydroquinoline-2-carboxylic acid (**6a**) and 3-((6,7-dimethoxy-3,4-dihydroisoquinolin-2(1H)-yl)methyl)-4-oxo-1,4-dihydroquinoline-2-carboxylic acid (**6b**).

**Scheme 2:**
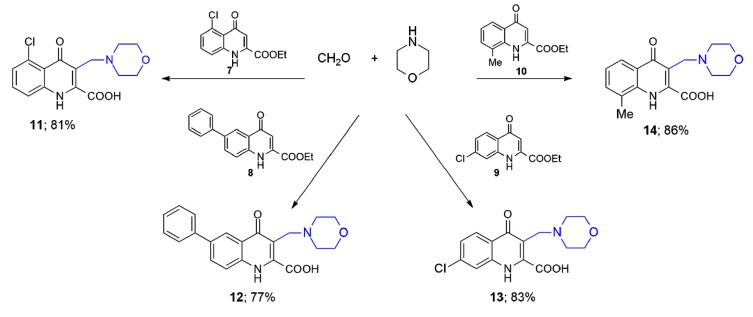
Syntheses of 5-, 6-, 7-, and 8-substituted KYNA Mannich derivatives.

To test the effect of substituent at 5-, 6-, 7- or 8-positions morpholine as a representative secondary cyclic amine was selected. First, the initial KYNA analogues (**7**–**10**) were synthesised with the Conrad–Limpach method applying the following optimization: a) the intermediate was purified by column chromatography using n-hexane:EtOAc as eluent and b) ring closure was carried out utilizing 1,2-dichlorobenzene instead of diphenyl ether used in the literature [8]. Reactions leading to the formation of **11**–**14** were performed starting from the corresponding ethyl ester and morpholine in the presence of formaldehyde (Scheme 2). It can be concluded that aryl/alkyl substituents at position 6 or 8, and the halogen at position 5 or 7 have no significant influence on the substitution at position 3.

**Scheme 3:**
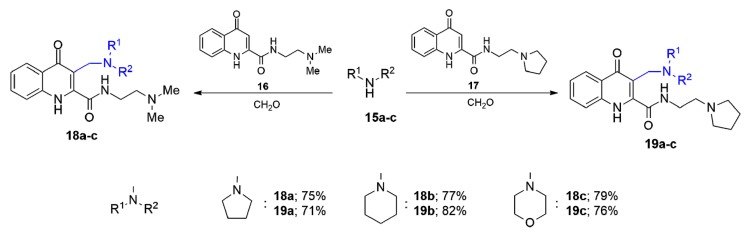
Syntheses of C-3 aminoalkylated KYNA amides.

Since KYNA amides **16** and **17**—containing cationic side-chain—proved to be the most effective analogues [27,28], our attention focused on the substitution of these compounds at position 3 via the *m*Mr. In this reaction, different secondary amines such as pyrrolidine, piperidine, and morpholine were tested. Substrates *N*-(2-(dimethylamino)ethyl)-4-oxo-1,4-dihydroquinoline-2-carboxamide (**16**) and 4-oxo-*N*-(2-(pyrrolidin-1-yl)ethyl)-1,4-dihydroquinoline-2-carboxamide (**17**), synthesised according to a literature method [21], were aminoalkylated with **15a**–**c** in the presence of formaldehyde, resulting in aminoalkylated KYNA derivatives **18a**–**c** and **19a**–**c**, respectively (Scheme 3).

Summarizing these results and those of a previous study [29], the synthesis of this type of KYNA derivatives containing an amide moiety at position 2 and an aminoalkyl group at position 3 can be achieved by applying two different synthetic pathways: amidation followed by aminoalkylation (route A) and a reverse reaction sequence (route B). Our results show that route A, that is amidation followed by aminoalkylation is more favorable, since it affords higher yields. For further investigation, the synthesis of **21** as representative aminoalkylated amide has been selected. 

**Scheme 4:**
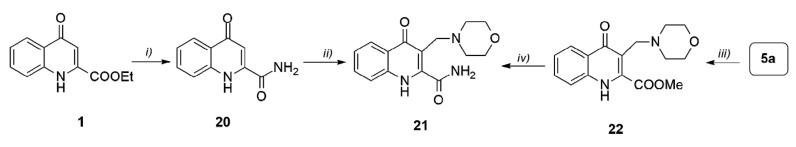
Synthetic pathways to obtain aminoalkylated KYNA amide **21**. route (A) (i) NH_3_/MeOH, 72%; (ii) morpholine, CH_2_O, 87%; route (B) (iii) CH_2_N_2_, 67%; (iv) NH_3_/MeOH, 88%.

A comparison of the overall yields to obtain **21** by using the two approaches shows that amidation followed by aminoalkylation (route A) resulted in the formation of the desired compounds in slightly higher yield (Scheme 4).

In the frame of our previous work, KYNA derivatives containing cationic centers (**5a**, **16**, **18c**, **17**, **19c**) have been surveyed in blood-brain-barrier models. It was concluded that **5a, 18c,** and **19c** have higher permeability towards the brain compared with amides **16** and **17**. In the case of the permeability measured from the brain to the outer compartment, compounds **5a** and **18c** showed the most promising results [P1]. Further investigation of the transport mechanism is still in progress.

**Figure 1:**
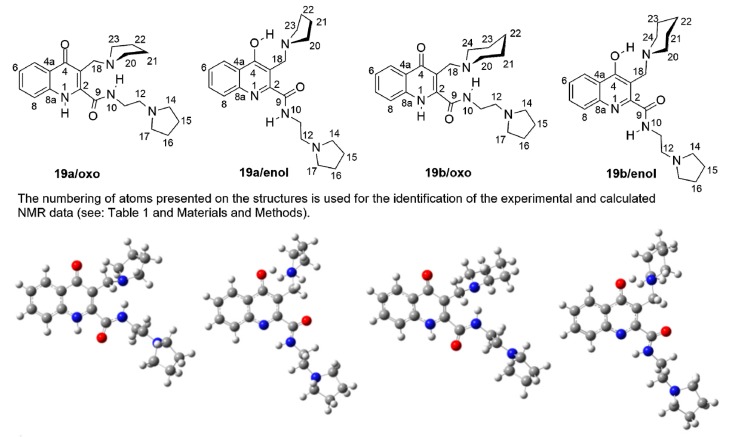
Optimised structures of the oxo- and enol forms of representative KYNA amides **19a**,**b** with the numbering of atoms used for the assignment of the experimental and calculated NMR data.

Finally, on two selected aminoalkylated KYNA amides **19a** and **19b** with closely related structures we studied the possible oxo-enol tautomerisation associated with the intramolecular hydrogen-bond framework that might exert significant influence on the intermolecular binding properties thus, on the drug-like character of these highly fuctionalised heterocycles. On the basis of the cross-peak correlations discernible in the 2D-COSY-, 2D-HSQC- and 2D-HMBC spectra, complete assignment of ^1^H- and ^13^C-NMR data was performed for the two investigated compounds assumed first to have oxo structures (Figure 1). The highly similar ^1^H- and ^13^C-NMR spectra of these compounds registered in DMSO-*d*_6_ solution refer to their practically identical structural features also including oxo-enol tautomerism. One of the two downfield broadened signals could be identified as 10-NH amide resonance coupled with the quartet originated from the adjacent CH_2_ group as disclosed by a COSY measurement carried out for **19b** that gives somewhat sharper signals than does **19a**. Regarding tautomerisation, it is of pronounced importance that in the NOESY spectrum of **19b** weak, but clearly discernible cross peaks connecting H-8 doublet with the most downfielded broadened signal (separated from the amide 10-NH signal by ca. 0.4 ppm) strongly suggest the presence of the oxo tautomer. Thus, this signal must be originated from the skeletal 1-NH proton being in the proximity of H-8. In order to get an additional support for the exclusive presence or dominance of the oxo tautomers over the enol forms, theoretical ^1^H- and ^13^C-NMR chemical shifts were calculated for both **19a**,**b** and their enol forms (Figure 1, Table 1) by GIAO method [30] using B3LYP fuctional [31,32,33] and the extended 6-311++G(2d,p) basis set [34]. The calculations were carried out on the structures optimised by the same functional with 6-31+G(d,p) basis set [35] and IEFPCM solvent model [36] employing dielectric constant of DMSO (ε = 46.7) that represents the conditions of NMR measurements. The comparision of the diagnostic chemical shifts of the skeletal and directly attached atoms calculated for the tautomer pairs with the corresponding experimental ^1^H- and ^13^C-NMR chemical shifts (Table 1) supports at least the dominance of the oxo tautomers over the enol forms. Finally, the relative thermodynamic stability obtained for the tautomeric pairs by B3LYP/6-31 + G(d,p) method also confirm the dominance of the oxo tautomers over the enol counterparts [∆G(**19a**/**enol** − **19a**/**oxo**) = +0.57 kcal/mol, ∆G(**19b**/**enol** − **19b**/**oxo**) = +0.59 kcal/mol].

## 3. Conclusions

A series of novel C-3 substituted kynurenic acid derivatives were synthesised by using the modified Mannich reaction. Aminoalkylations were achieved starting from primary or secondary amines, using formaldehyde as the representative aldehyde component. In addition, the applicability of benzaldehyde was also proven. Among the amine components, cyclic secondary amines such as morpholine, piperidine, or *N*-methylpiperazine were used. The limitations of the reaction were examined on the synthesis of different KYNA derivatives containing phenyl, chloro, or methyl substituents in ring B. The modified Mannich reaction was further tested starting from KYNA amides containing cationic center at the side-chain. In the case of **21** both sequences proceeding via aminoalkylation followed by amidation or amidation followed by aminoalkylation, were investigated. Amidation followed by aminoalkylation resulting in **21** was found to give a somewhat higher yield. By means of ^1^H- and ^13^C-NMR spectroscopy including NOESY measurement it was established that two representative aminoalkylated products feature quinolin-4(1H)-one skeletal structure rather than quinolin-4-ol tautomer form. The dominance of the oxo form over the enol counterpart, also supported by DFT modelling studies, can certainly be stated for all the KYNA amides presented in this contribution.

## 4. Materials and Methods

The ^1^H and ^13^C-NMR spectra were recorded in DMSO-*d*_6_, CDCl_3_ and D_2_O solutions in 5 mm tubes at room temperature (RT), on a Bruker DRX-500 spectrometer (Bruker Biospin, Karlsruhe, Baden Württemberg, Germany) at 500 (1H) and 125 (13C) MHz, with the deuterium signal of the solvent as the lock and TMS as internal standard (1H, 13C). The 2D-COSY, NOESY, HSQC, and HMBC spectra of **19a** and **19b** were obtained by using the standard Bruker pulse programs (cosygpppqf (2D COSY with gradient pulses for selection and purge pulses before relaxation delay d1) for COSY, noesygpphpp (2D phase sensitive NOESY with gradient pulses in mixing time and purge pulses before relaxation delay d1 for NOESY), hsqcetgp (2D phase sensitive HSQC using Echo/Antiecho-TPPI gradient selection with decoupling during acquisition and using trim pulses in inept transfer) for HSQC and hmbcgpndqf (2D H-1/X HMBC optimised on long range couplings, no decoupling during acquisition using gradient pulses for selection) for HMBC, Bruker Biospin, Karlsruhe, Baden Württemberg, Germany). All calculations were carried out by using Gaussian 09 software package [37]. The optimised structures are available from the authors.

Melting points were determined on a Hinotek X-4 melting point apparatus. Elemental analyses were performed with a Perkin-Elmer 2400 CHNS elemental analyser. Merck Kieselgel 60F_254_ plates were used for TLC.

The synthesis of compounds **1** [38] and **5a** [29] was based on the method known from the literature.

^1^H- and ^13^C-NMR chemical shifts were calculated for both **19a**,**b** and their enol forms (Figure 1, Table 1) by GIAO method [30] using B3LYP functional [31,32,33] and the extended 6-311++G(2d,p) basis set [34]. The calculations were carried out on the structures optimised by the same functional with 6-31+G(d,p) basis set [35] and IEFPCM solvent model [36] employing dielectric constant of DMSO (ε = 46.7) that represents the conditions of NMR measurements.

### 4.1. General Procedure for the Synthesis of C-3 Substituted Kynurenic Acid Derivatives (***2**, **3**, **4a**,**b**, **5b**, **5c**, **6a**,**b***)

4-Oxo-1,4-dihydroquinoline-2-carboxylic acid ethyl ester (**1**, 326 mg, 1.5 mmol), primary or secondary amine (2.0 mmol), and aldehyde (3.0 mmol) were placed in a 50 mL round-bottom flask. The mixture was treated at reflux temperature in 1,4-dioxane (30 mL) for 0.5–4 h. After the evaporation of the solvent the residue was dissolved in H_2_O (40 mL) and extracted with DCM (3 × 30 mL). The collected organic phases were dried (Na_2_SO_4_), the solvent was evaporated, and the residue was crystallised from a n-hexane:EtOAc (95:5) mixture (10 mL).

#### 4.1.1. 3-(((2-Morpholinoethyl)amino)methyl)-4-oxo-1,4-dihydroquinoline-2-carboxylic acid (**2**)

Preparation according to general procedure, using 2-morpholinoethylamine (260 mg, 2.0 mmol) and aqueous formaldehyde (22%, 410 mg, 3.0 mmol); reflux time: 1 h. Yield: 473 mg (78%); M.p. 300–303 °C. ^1^H NMR (DMSO-*d*_6_); 3.07–3.2 (2H, m); 3.47–3,56 (2H, m); 3.79 (2H, t, *J* = 12.2 Hz); 3.92–4.04 (4H, m); 4.47 (2H, s); 7.4 (1H, t, *J* = 7.5 Hz); 7.73 (1H, t, *J* = 8.1 Hz); 7.82 (1H, d, *J* = 8.3 Hz); 8.20 (1H, d, *J* = 8.0 Hz); 10.86 (1H, br ~s); ^13^C NMR (DMSO-*d*_6_); 37.5; 47.8; 51.9; 54.2; 63.8; 120.3; 121.6; 124.5; 125.8; 127.1; 133.2; 141.2; 141.3; 165.4; 174.0; (Figures S1 and S2 in Supplementary Materials).

#### 4.1.2. 3-(((2-(Dimethylamino)ethyl)amino)(phenyl)methyl)-4-oxo-1,4-dihydroquinoline-2-carboxylic acid (**3**)

Preparation according to general procedure, using *N*,*N*-dimethylethylene diamine (176 mg, 2.0 mmol) and benzaldehyde (318 mg, 3.0 mmol); reflux time: 0.5 h. Yield: 487 mg (74%); M.p. 193–195 °C. ^1^H NMR (DMSO-*d*_6_); 2.81 (6H, s); 2.85–2.94 (1H, m); 3.12-3.22 (1H, m); 3.44–3.54 (1H, m); 4.12–4.23 (1H, m); 5.84 (1H, s); 7.30–7.41 (6H, m); 7.73 (1H, t, *J* = 7.8 Hz); 7.86 (1H, d, *J* = 8.5 Hz); 8.07 (1H, d, *J* = 8.1 Hz); 10.01 (1H, br ~s); 12.85 (1H, br ~s); ^13^C NMR (DMSO-*d*_6_); 35.7; 42.1; 44.2; 54.7; 61.2; 120.3; 124.6; 125.0; 125.8; 127.8; 128.9; 129.4; 129.7; 133.2; 136.0; 141.1; 141.2; 165.2; 173.3; (Figures S3 and S4).

#### 4.1.3. 3-((Dimethylamino)methyl)-4-oxo-1,4-dihydroquinoline-2-carboxylic acid (**4a**)

Preparation according to general procedure, using dimethylamine (90 mg, 2.0 mmol) and aqueous formaldehyde (22%, 410 mg, 3.0 mmol); reflux time: 1 h; Yield: 303 mg (82%); M.p. 246–249 °C. ^1^H NMR (DMSO-*d*_6_); 2.75 (6H, s); 4.36 (2H, s); 7.36 (1H, t, *J* = 7.7 Hz); 7.68 (1H, t, *J* = 7.8 Hz); 8.01 (1H, d, *J* = 7.2 Hz); 8.12 (1H, d, *J* = 7.6 Hz); 11.05 (1H, br ~s); 11.75 (1H, br ~s); ^13^C NMR (DMSO-*d*_6_); 42.0; 52.6; 109.3; 120.3; 124.6; 125.0; 126.0; 133.0; 139.7; 149.1; 164.7; 178.0; (Figures S5 and S6).

#### 4.1.4. 3-((Benzyl(methyl)amino)methyl)-4-oxo-1,4-dihydroquinoline-2-carboxylic acid (**4b**)

Preparation according to general procedure, using N-benzylmethylamine (242 mg, 2.0 mmol) and aqueous formaldehyde (22%, 410 mg, 3.0 mmol); reflux time: 2 h. Yield: 411 mg (85%); M.p. 235–237 °C. ^1^H NMR (DMSO-*d*_6_); 2.43–2.49 (4H, m); 4.16–4.58 (5H, m); 7.35 (1H, t, *J* = 7.3 Hz); 7.41–7.55 (5H, m); 7.67 (1H, t, *J* = 7.6 Hz); 7.99 (1H, d, *J* = 8.4 Hz); 8.10 (1H, d, *J* = 8.0 Hz); 11.82 (1H, br ~s); 12.75 (1H, br ~s); ^13^C NMR (DMSO-*d*_6_); 37.0; 50.5; 57.7; 108.7; 119.9; 124.3; 124.5; 125.6; 129.4; 129.7; 131.0; 131.7; 132.6; 139.4; 148.6; 164.9; 177.5; (Figures S7 and S8).

#### 4.1.5. 4-Oxo-3-(piperidin-1-ylmethyl)-1,4-dihydroquinoline-2-carboxylic acid (**5b**)

Preparation according to general procedure, using piperidine (170 mg, 2.0 mmol) and aqueous formaldehyde (22%, 410 mg, 3.0 mmol); reflux time: 1 h. Yield: 353 mg (73%); M.p. 215–217 °C. ^1^H NMR (DMSO-*d*_6_); 1.38–1.52 (1H, m); 1.59–1.77 (3H, m); 1.78–1.86 (2H, m); 3.07 (2H, t, *J* = 12.63 Hz); 3.34 (2H, d, *J* = 11.93 Hz); 4.50 (2H, s); 7.44 (1H, t, *J* = 7.6 Hz); 7.76 (1H, t, *J* = 7.8 Hz); 8.05 (1H, d, *J* = 8.5 Hz); 8.14 (1H, d, *J* = 8.0 Hz); 9.35 (1H, br ~s); 12.20 (1H, br ~s); ^13^C NMR (DMSO-*d*_6_); 22.0; 23.1; 51.6; 53.3; 111.4; 120.5; 125.2; 125.6; 125.9; 133.8; 139.9; 142.6; 164.6; 178.2; (Figures S9 and S10).

#### 4.1.6. 3-((4-Methylpiperazin-1-yl)methyl)-4-oxo-1,4-dihydroquinoline-2-carboxylic acid (**5c**)

Preparation according to general procedure, using 1-methylpiperazine (200 mg, 2.0 mmol) and aqueous formaldehyde (22%, 410 mg, 3.0 mmol); reflux time: 0.5 h. Yield: 352 mg (78%); M.p. 229–231 °C. ^1^H NMR (DMSO-*d*_6_); 2.22 (3H, s); 2.74–3.11 (4H, m); 3.35–3.41 (4H, m); 4.35 (2H, s); 7.34 (1H, t, *J* = 7.4 Hz); 7.66 (1H, t, *J* = 7.6 Hz); 7.97 (1H, d, *J* = 8.4 Hz); 8.09 (1H, d, *J* = 8.0 Hz); 11.75 (1H, br ~s); ^13^C NMR (DMSO-*d*_6_); 45.8; 50.1; 50.3; 52.7; 108.7; 120.3; 124.6; 124.9; 126.0; 133.0; 139.8; 148.9; 165.2; 177.9; (Figures S11 and S12).

#### 4.1.7. 3-((3,4-Dihydroisoquinolin-2(1H)-yl)methyl)-4-oxo-1,4-dihydroquinoline-2-carboxylic acid (**6a**)

Preparation according to general procedure, using 1,2,3,4-tetrahydroisoquinoline (266 mg, 2.0 mmol) and aqueous formaldehyde (22%, 410 mg, 3.0 mmol); reflux time: 3 h. Yield: 406 mg (81%); M.p. 275–278 °C. ^1^H NMR (DMSO-*d*_6_); 3.01–3.16 (2H, m); 3.36–3.57 (2H, m); 4.32–4.46 (2H, m); 4.51 (2H, s); 7.17–7.32 (4H, m); 7.36 (1H, t, *J* = 7.6 Hz); 7.68 (1H, t, *J* = 7.4 Hz); 8.00 (1H, d, *J* = 8.4 Hz); 8.12 (1H, d, *J* = 8.0 Hz); 11.77 (1H, br ~s); 13.06 (1H, br ~s); ^13^C NMR (DMSO-*d*_6_); 25.9; 47.5; 49.8; 51.7; 108.9; 120.4; 124.7; 124.9; 126.0; 127.5; 127.8; 128.5; 129.5; 129.7; 132.4; 133.0; 139.8; 148.8; 165.2; 178.0; (Figures S13 and S14).

#### 4.1.8. 3-((6,7-Dimethoxy-3,4-dihydroisoquinolin-2(1H)-yl)methyl)-4-oxo-1,4-dihydroquinoline-2-carboxylic acid (**6b**)

Preparation according to general procedure, using 6,7-dimethoxy-1,2,3,4-tetrahydroisoquinoline (382 mg, 2.0 mmol) and aqueous formaldehyde (22%, 410 mg, 3.0 mmol); reflux time: 4 h. The crystals were purified in EtOH (10 mL). Yield: 355 mg (60%); M.p. 248–250 °C. ^1^H NMR (DMSO-*d*_6_); 2.90−3.08 (2H, m); 3.50−3.60 (2H, m); 3.69 (3H, s); 3.74 (3H, s); 4.16–4.37 (2H, m); 4.49 (2H, s); 6.78 (1H, s); 6.81 (1H, s); 7.37 (1H, t, *J* = 7.7 Hz); 7.67 (1H, t, *J* = 7.4 Hz); 8.00 (1H, d, *J* = 8.8 Hz); 8.13 (1H, d, *J* = 7.8 Hz); 11.75 (1H, br ~s); 13.03 (1H, br ~s); ^13^C NMR (DMSO-*d*_6_); 25.3; 47.4; 49.4; 50.8; 56.0; 63.1; 108.5; 110.4; 112.0; 119.9; 120.8; 123.7; 124.3; 124.5; 125.6; 132.7; 139.4; 148.1; 148.4; 148.8; 164.8; 177.6; (Figures S15 and S16).

### 4.2. General Procedure for the Synthesis of 5-, 6-, 7-, and 8-Substituted Kynurenic Acid Derivatives (***7**–**10***)

Diethyl acetylenedicarboxylate (510 mg, 3.0 mmol) and 2.0 mmol of the corresponding aniline derivatives (4-aminobiphenyl, 338 mg; 3-chloroaniline, 255 mg; 2-methylaniline 214 mg) were dissolved in EtOH (60 mL). The mixture was treated at reflux temperature for 1–8 h. After the evaporation of the solvent, the intermediate enamine was purified using column-chromatography (eluent: n-hexane:EtOAc 1:1). After the purification the intermediate was mixed with 1,2-dichlorobenzene (40 mL) and then was treated at reflux temperature for 10–24 h. After the solvent was removed by evaporation, the residue was crystallised from Et_2_O:EtOAc 95:5 mixture (10 mL).

#### 4.2.1. Ethyl 5-chloro-4-oxo-1,4-dihydroquinoline-2-carboxylate (**7**)

Reflux time: 8 h (for enamine formation), 24 h (for ring closure). The crystals were recrystallised from EtOAc (5 mL). Yield: 131 mg (26%); M.p. 200–205, (lit [39]: 197–198 °C). ^1^H NMR (DMSO-*d*_6_); 1.37 (3H, t, *J* = 7. Hz); 4.42 (2H, q, *J* = 7.2 Hz); 6.58 (1H, s); 7.32 (1H, d, *J* = 7.3 Hz); 7.59 (1H, t, *J* = 8.1 Hz); 7.91 (1H, d, *J* = 8.4 Hz); 11.93 (1H, br ~s); ^13^C NMR (DMSO-*d*_6_); 14.4; 63.1; 112.8; 119.4; 122.4; 126.9; 132.3; 132.7; 137.4; 143.2; 162.4; 194.2; (Figures S17 and S18).

#### 4.2.2. Ethyl 4-oxo-6-phenyl-1,4-dihydroquinoline-2-carboxylate (**8**)

Reflux time: 1 h (for enamine formation) and 10 h (for ring closure). The crystals were recrystallised from EtOAc (8 mL) Yield: 422 mg (72%); M.p. 263–265 °C, (lit [40]: 261–261.5 °C). ^1^H NMR (CDCl_3_); 1.39 (3H, t, *J* = 7.2 Hz); 4.45 (2H, q, *J* = 7.0 Hz); 6.68 (1H, s); 7.40 (1H, t, *J* = 7.1 Hz); 7.50 (2H, t, *J* = 7.5 Hz); 7.74 (2H, d, *J* = 7.5 Hz); 8.02–8.10 (2H, m); 12.10 (1H, br ~s); ^13^C NMR (CDCl_3_); 14.1; 63.4; 111.3; 118.8; 123.9; 126.4; 127.1; 127.8; 129.0; 132.4; 136.6; 137.8; 138.3; 139.5; 162.8; 179.3; (Figures S19 and S20).

#### 4.2.3. Ethyl 7-chloro-4-oxo-1,4-dihydroquinoline-2-carboxylate (**9**)

Reflux time: 8 h (for enamine formation), 20 h (for ring closure). Yield: 211 mg (42%); M.p. 258–259 °C, (lit [39]: 250–251 °C). ^1^H NMR (DMSO-*d*_6_); 1.37 (3H, t, *J* = 7.0 Hz); 4.43 (2H, q, *J* = 7.2 Hz); 6.65 (1H, s); 7.39 (1H, d, *J* = 8.0 Hz); 8.01 (1H, s); 8.07 (1H, d, *J* = 8.8 Hz); 12.09 (1H, br ~s); ^13^C NMR (DMSO-*d*_6_); 14.4; 63.3; 111.2; 119.1; 124.9; 124.9; 127.6; 137.7; 138.8; 141.2; 162.4; 177.5; (Figures S21 and S22).

#### 4.2.4. Ethyl 4-oxo-8-methyl-1,4-dihydroquinoline-2-carboxylate (**10**)

Reflux time: 3 h (for enamine formation), 14 h (for ring closure). Yield: 337 mg (73%); M.p. 138–143 °C, (lit [41]: 139–139.5 °C). ^1^H NMR (CDCl_3_); 1.46 (3H, t, *J* = 7,1 Hz) 2.58 (3H, s); 4.51 (2H, q, *J* = 7.0 Hz); 7.11 (1H, s); 7.31 (1H, t, *J* = 7.6 Hz); 7.53 (1H, d, *J* = 7.0 Hz); 8.23 (1H, d, *J* = 8.2 Hz); 9.03 (1H, br ~s); ^13^C NMR (CDCl_3_); 14.0; 16.5; 63.4; 111.4; 124.3; 124.4; 125.2; 126.3; 133.8; 136.1; 137.8; 163.1; 179.8; (Figures S23 and S24).

### 4.3. General Procedure for the Synthesis of B Ring Substituted C-3 Morpholinomethyl Kynurenic Acid Derivatives (***11**–**14***)

The correspondingly substituted kynurenic acid ethyl esters (**7**–**10** 1.5 mmol), morpholine (175 mg, 2.0 mmol), and aqueous formaldehyde (22%, 410 mg, 3.0 mmol) were mixed in 1,4-dioxane (30 mL) and then hetead at reflux temperature for 6–12 h. After the evaporation of the solvent the residue was dissolved in H_2_O (40 mL), and extracted with DCM (3 × 30 mL). The collected organic phases were dried (Na_2_SO_4_), the solvent was removed by evaporation, and the residue was crystallised from n- hexane:EtOAc 95:5 mixture (10 mL).

#### 4.3.1. 5-Chloro-3-(morpholinomethyl)-4-oxo-1,4-dihydroquinoline-2-carboxylic acid (**11**)

Preparation according to general procedure, using ethyl 5-chloro-4-oxo-1,4-dihydroquinoline-2-carboxylate (**7**, 377 mg, 1.5 mmol; reflux time: 6 h. The crystals were purified in EtOH (10 mL). Yield: 368 mg (70%); M.p. 265–270 °C (decomposition). ^1^H NMR (DMSO-*d*_6_); 2.97–3.32 (4H, m); 3.48–3.77 (2H, m); 3.78–4.10 (2H, m); 4.35 (2H, s); 7.30 (1H, d, *J* = 7.9 Hz); 7.55 (1H, t, *J* = 8.12 Hz); 7.96 (1H, d, *J* = 8.5 Hz); 11,69 (1H, br ~s); 12.60 (1H, br ~s); ^13^C NMR (DMSO-*d*_6_); 50.1; 50.6; 64.3; 109.9; 119.4; 120.6; 126.8; 132.4; 132.4; 137.7; 142.0; 147.5; 164.4; 177.0; (Figures S25 and S26).

#### 4.3.2. 3-(Morpholinomethyl)-4-oxo-6-phenyl-1,4-dihydroquinoline-2-carboxylic acid (**12**)

Preparation according to general procedure, using ethyl 4-oxo-6-phenyl-1,4-dihydroquinoline-2-carboxylate (**9**, 440 mg, 1.5 mmol); reflux time: 12 h. Yield: 420 mg (77%); M.p. 225–227 °C. ^1^H NMR (DMSO-*d*_6_); 3.02–3.20 (2H, m); 3.20–3.37 (2H, m); 3.52–3.71 (2H, m); 3.89–4.08 (2H, m); 4.44 (2H, s); 7.40 (1H, t, *J* = 7.2 Hz); 7.51 (2H, t, *J* = 7.9 Hz); 7.74 (2H, d, *J* = 7.7 Hz); 8.03 (1H, d, *J* = 8.6 Hz); 8.10 (1H, d, *J* = 8.8 Hz); 8.34 (1H, s); 11.94 (1H, br ~s); 12.72 (1H, br ~s); ^13^C NMR (DMSO-*d*_6_); 50.0; 50.6; 64.3; 108.3; 120.8; 122.8; 124.7; 127.1; 128.1; 129.6; 131.4; 136.1; 138.8; 139.8; 148.4; 164.8; 177.6; (Figures S27 and S28).

#### 4.3.3. 7-Chloro-3-(morpholinomethyl)-4-oxo-1,4-dihydroquinoline-2-carboxylic acid (**13**)

Preparation according to general procedure, using ethyl 7-chloro-4-oxo-1,4-dihydroquinoline-2-carboxylate (**11**, 377 mg, 1.5 mmol); reflux time: 6 h. Yield: 402 mg (83%); M.p. 278–281 °C. ^1^H NMR (DMSO-*d*_6_); 2.97–3.35 (4H, m); 3.51–3.71 (2H, m); 3.81–4.03 (2H, m); 4.39 (2H, s); 7.36 (1H, d, *J* = 8.7 Hz); 8.06-8.11 (2H, m); 11.85 (1H, br ~s); 12.57 (1H, br ~s); ^13^C NMR (DMSO-*d*_6_); 29.5; 50.2; 50.5; 64.4; 66.0; 119.0; 123.1; 124.6; 127.9; 137.2; 140.1; 148.9; 164.4; 177.2; (Figures S29 and S30).

#### 4.3.4. 8-Methyl-3-(morpholinomethyl)-4-oxo-1,4-dihydroquinoline-2-carboxylic acid (**14**)

Preparation according to general procedure, using ethyl 4-oxo-8-methyl-1,4-dihydroquinoline-2-carboxylate (**13**, 347 mg, 1.5 mmol); reflux time: 8 h. Yield: 390 mg (86%); M.p. 255–258 °C. ^1^H NMR (DMSO-*d*_6_); 2.54 (3H, s); 3.12–3.28 (4H, m); 3.63–3.95 (4H, m); 4.51 (2H, s); 7.29 (1H, t, *J* = 7.8 Hz); 7.58 (1H, d, *J* = 7.2 Hz); 7.99 (1H, d, *J* = 8.1 Hz); 10.22 (1H, br ~s); ^13^C NMR (DMSO-*d*_6_); 16.9; 50,8; 50,9; 64.5; 109.2; 124.3; 124.6; 124.8; 127.1; 134.0; 137.4; 146.4; 164.4; 178.6; (Figures S31 and S32).

### 4.4. General Procedure for the Synthesis of C-3 Morpholinomethyl Kynurenic Acid Amides (***18a**–**c**, **19a**–**c***)

The corresponding kynurenic acid amides (**16**, **17**, 1.5 mmol), and secondary amines (**15a**–**c**, 2.0 mmol), were mixed with formaldehyde (22%, 410 mg, 3.0 mmol) in a 50 mL round-bottom flask. The mixture was heated at reflux temperature in 1,4-dioxane (30 mL) for 1–8 h. After the evaporation of the solvent the residue was crystallised from Et_2_O (10 mL).

#### 4.4.1. *N*-(2-(Dimethylamino)ethyl)-4-oxo-3-(pyrrolidin-1-ylmethyl)-1,4-dihydroquinoline-2-carboxamide (**18a**)

Preparation according to general procedure, using *N*-(2-(dimethylamino)ethyl)-4-oxo-1,4-dihydroquinoline-2-carboxamide (**16**, 389 mg, 1,5 mmol), pyrrolidine (**15a** 142 mg, 2.0 mmol); reflux time: 1 h. Yield: 385 mg (75%); M.p. > 350 °C. ^1^H NMR (D_2_O); 2.12 (4H, m); 2.38 (6H, s); 2.76 (2H, t, *J* = 5.5 Hz); 3.41 (4H, m); 3.63 (2H, t, *J* = 6.3 Hz); 4.48 (2H, s) 7.52 (1H, t, *J* = 7.2 Hz); 7.75 (1H, t, *J* = 6.8 Hz); 7.82 (1H, d, *J* = 8.1 Hz); 8.23 (1H, d, *J* = 8.0 Hz); ^13^C NMR (D_2_O); 22.8; 36.9; 43.9; 51.6; 53.3; 56.8; 107.5; 123.9; 125.1; 126.0; 127.2; 131.0; 147.9; 152.6; 169.9; 174.5; (Figures S33 and S34).

#### 4.4.2. *N*-(2-(Dimethylamino)ethyl)-4-oxo-3-(piperidin-1-ylmethyl)-1,4-dihydroquinoline-2-carboxamide (**18b**)

Preparation according to general procedure, using *N*-(2-(dimethylamino)ethyl)-4-oxo-1,4-dihydroquinoline-2-carboxamide (**16**, 389 mg, 1,5 mmol) and piperidine (**15b** 170 mg, 2.0 mmol); reflux time: 1 h. Yield: 412 mg (77%); M.p. > 350 °C. ^1^H NMR (D_2_O); 1.62–1.76 (2H, m); 1.76–1.91 (4H, m); 2.38 (6H, s); 2.75 (2H, t, *J* = 6.0 Hz); 3.24 (4H, m); 3.62 (2H, t, *J* = 6.0 Hz); 4.40 (2H, s); 7.53 (1H, t, *J* = 7.2 Hz); 7.75 (1H, t, *J* = 7.2 Hz); 7.82 (1H, d, *J* = 8.5 Hz); 8.22 (1H, d, *J* = 8.2 Hz); ^13^C NMR (D_2_O); 21.5; 23.2; 36.9; 43.9; 52.3; 53.4; 56.8; 106.2; 123.8; 125.2; 125.8; 127.2; 131.1; 147.8, 152.9; 170.1; 174.6; (Figures S35 and S36).

#### 4.4.3. *N*-(2-(Dimethylamino)ethyl)-3-(morpholinomethyl)-4-oxo-1,4-dihydroquinoline-2-carboxamide (**18c**)

Preparation according to general procedure, using *N*-(2-(dimethylamino)ethyl)-4-oxo-1,4-dihydroquinoline-2-carboxamide (**16**, 389 mg, 1,5 mmol) and morpholine (**15c** 174 mg, 2.0 mmol); reflux time: 8 h. Yield: 425 mg (79%); M.p. > 350 °C. ^1^H NMR (DMSO-*d*_6_); 2.19 (6H, s); 2.41–2.48 (6H, m); 3.45–3.52 (2H, m); 3.53–3.59 (4H, m); 3.61 (2H, s) 7.32 (1H, t, *J* = 7.3 Hz); 7.64 (1H, t, *J* = 7.7 Hz); 7.86 (1H, d, *J* = 8.3 Hz); 8.08 (1H, d, *J* = 8.1 Hz); 10.63 (1H, br ~s); ^13^C NMR (DMSO-*d*_6_); 38.4; 46.0; 51.1; 52.6; 59.1; 67.0; 113.7; 120.4; 124.4; 124.8; 126.0; 131.0; 132.8; 140.5; 163.9; 177.8; (Figures S37 and S38).

#### 4.4.4. 4-Oxo-*N*-(2-(pyrrolidin-1-yl)ethyl)-3-(pyrrolidin-1-ylmethyl)-1,4-dihydroquinoline-2-carboxamide (**19a**)

Preparation according to general procedure, using 4-oxo-*N*-(2-(pyrrolidin-1-yl)ethyl)-1,4-dihydroquinoline-2-carboxamide (**17**, 428 mg, 1,5 mmol) and pyrrolidine (**15a**, 142 mg, 2.0 mmol); reflux time: 1 h. Yield: 392 mg (71%); M.p. > 350 °C. ^1^H NMR (DMSO-*d*_6_); 1.64 (4H, br ~s, H-15,16); 1.67 (4H, br ~s, H-21,22); 2.42 (4H, br ~s, H-14,17); 2.54 (4H, br ~s, H-20,23); 2.55 (2H, t, *J* = 5.8 Hz, H-12); 3.44 (2H, qa, *J* = 5.8 Hz, H-11); 3.68 (2H, br ~s, H-18); 7.26 (1H, d, *J* = 7.8 Hz, H-6); 7.58 (1H, br ~t, *J* = 8 Hz, H-7); 7.81 (1H, br ~d, *J* = 8 Hz, H-8); 8.05 (1H, br d, *J* = 7.8 Hz, H-5); 11.30 (1H, br ~s, 10-NH); 11.64 (1H, br ~s, 1-NH); ^13^C NMR (DMSO-*d*_6_); 23.6 (two coalesced lines, C-15,16,21,22); 39.4 (merged in the septet signal of the solvent, C-11); 52.2 (C-20,23); 47.5 (C-18); 52.2 (C-20,23); 54.1 (C-14,17); 55.2 (C-12); 115.1 (C-3); 119.6 (two coalesced lines, C-4a,8); 123.9 (C-6); 125.6 (C-5); 132.5 (C-7); 139.6 (C-8a); 143.5 (C-2); 163.3 (C-9); 177.3 (C-4); (Figures S39–S41).

#### 4.4.5. 4-Oxo-3-(piperidin-1-ylmethyl)-*N*-(2-(pyrrolidin-1-yl)ethyl)-1,4-dihydroquinoline-2-carboxamide (**19b**)

Preparation according to general procedure, using 4-oxo-*N*-(2-(pyrrolidin-1-yl)ethyl)-1,4-dihydroquinoline-2-carboxamide (**17**, 428 mg, 1,5 mmol) and piperidine (**15b**, 170 mg, 2.0 mmol); reflux time: 1 h. Yield: 470 mg (82%); M.p. > 350 °C. ^1^H NMR (DMSO-*d*_6_); 1,38 (2H, br ~s, H-22); 1.44 (4H, br ~s, H-21,23); 1.63 (4H, br ~s, H-15,16); 2.40 (4H, br ~s, H-20,24); 2.43 (4H, br ~s, H-14,17); 2.57 (2H, t, *J* = 5.8 Hz, H-12); 3.44 (2H, qa, *J* = 5.8 Hz, H-11); 3.52 (2H, br ~s, H-18); 7.28 (1H, t, *J* = 7.8 Hz, H-6); 7.60 (1H, br ~t, *J* = 8 Hz, H-7); 7.84 (1H, br ~d, *J* = 8 Hz, H-8); 8.03 (1H, br d, *J* = 7.8 Hz, H-5); 11.30 (1H, br ~s, 10-NH); 11.69 (1H, br ~s, 1-NH); ^13^C NMR (DMSO-*d*_6_); 23.6 (C-15,16); 24.3 (C-22); 25.9 (C-21, 23); 39.4 (C-11); 50.8 (C-18); 52.5 (C-20,24); 54.2 (C-14,17); 55.4 (C-12); 114.2 (C-3); 119.6 (C-4a); 119.6 (C-8); 124.1 (C-6); 125.6 (C-5); 132.6 (C-7); 139.7 (C-8a); 143.9 (C-2); 163.5 (C-9); 177.8 (C-4); (Figures S42–S45).

#### 4.4.6. 4-Oxo-3-(morpholinomethyl)-*N*-(2-(pyrrolidin-1-yl)ethyl)-1,4-dihydroquinoline-2-carboxamide (**19c**)

Preparation according to general procedure, using 4-oxo-*N*-(2-(pyrrolidin-1-yl)ethyl)-1,4-dihydroquinoline-2-carboxamide (**17**, 428 mg, 1,5 mmol) and morpholine (**15c**, 174 mg, 2.0 mmol); reflux time: 8 h. Yield: 438 mg (76%); M.p. > 350 °C. ^1^H NMR (DMSO-*d*_6_); 1.63–1.73 (4H, m); 2.46–2.52 (6H, m); 2.63 (2H, t, *J* = 6.2 Hz); 3.48–3.63 (10H, m); 7.34 (1H, t, *J* = 7.1 Hz); 7.66 (1H, t, *J* = 7.2 Hz); 7.88 (1H, d, *J* = 8.0 Hz); 8.09 (1H, d, *J* = 8.0 Hz); 10.82 (1H, br ~s); 11.78 (1H, br ~s); ^13^C NMR (DMSO-*d*_6_); 23.6; 50.6; 52.1; 54.1; 55.4; 66.6; 113.4; 119.6; 124.2; 124.3; 125.7; 132.6; 139.7; 144.3; 163.2; 177.7; (Figures S46 and S47).

### 4.5. 4-Oxo-1,4-dihydroquinoline-2-carboxamide (***20***)

Ethyl 4-oxo-1,4-dihydroquinoline-2-carboxylate (**1**, 652 mg, 3.0 mmol) and NH_3_/MeOH (20%, 5 mL) were placed in a pressure-resistant vessel of 10 mL. The mixture was kept at 100 °C for 30 min with a CEM Discover SP microwave reactor. Following the removal of the solvent the residue was crystallised from Et_2_O (10 mL) and recrystallised from EtOH (8 mL).

Yield: 355 mg (63%); M.p. 300–303 °C (lit [42]: 295–297 °C). ^1^H NMR (DMSO-*d*_6_); 6.75 (1H, s); 7.33 (1H, t, *J* = 7.6 Hz); 7.67 (1H, t, *J* = 7.7 Hz); 7.96 (1H, d, *J* = 8.4 Hz); 8.03 (1H, s); 8.07 (1H, d, *J* = 8.2 Hz); 8.44 (1H, s) 11.69 (1H, br ~s); ^13^C NMR (DMSO-*d*_6_); 107.8; 120.0; 124.1; 125.1; 125.9; 132.7; 140.3; 141.6; 164.1; 178.4; (Figures S48 and S49).

### 4.6. Methyl 3-(morpholinomethyl)-4-oxo-1,4-dihydroquinoline-2-carboxylate (***22***)

In a 50 mL round-bottom flask 576 mg (2.0 mmol) 3-(morpholinomethyl)-4-oxo-1,4-dihydroquinoline-2-carboxylic acid (**5a**) was dissolved in 20 mL MeOH. The mixture was reacted with 1 mL Et_2_O solution of CH_2_N_2_ at room temperature for 5 h. Following the removal of the solvent the residue was crystallised from n-hexane:EtOAc (95:5; 10 mL).

Yield: 405 mg (67%); M.p. 122–123 °C. ^1^H NMR (DMSO-*d*_6_); 1.23 (3H, s); 2.99–3.19 (2H, m); 2.19–3.35 (2H, m); 3.50–3.69 (2H, m); 3.87–4.04 (2H, m); 4,40 (2H, s); 7.35 (1H, t, *J* = 7.5 Hz); 7.67 (1H, t, *J* = 7.8 Hz); 7.99 (1H, d, *J* = 8.3 Hz); 8.09 (1H, d, *J* = 8.1 Hz); 11.81 (1H, br ~s); 12.71 (1H, br ~s); ^13^C NMR (DMSO-*d*_6_); 29.5; 49.9; 50.6; 64.3; 108.1; 119.9; 124.3; 124.5; 125.5; 132.6; 139.4; 148.5; 164.8; 177.6; (Figures S52 and S53).

### 4.7. 3-(Morpholinomethyl)-4-oxo-1,4-dihydroquinoline-2-carboxamide (***21***)

Scheme 4: (ii): Carboxamide **20** (188 mg, 1.0 mmol), morpholine (174 mg, 2.0 mmol) and aqueous formaldehyde (22%, 410 mg, 3.0 mmol) were heated at reflux temperature in 1,4-dioxane for 3 h. After evaporation of the solvent the residue was crystallised with Et_2_O (10 mL). Yield A: 202 mg (87%); M.p. 144–145 °C.

Scheme 4: (iv): Methyl 3-(morpholinomethyl)-4-oxo-1,4-dihydroquinoline-2-carboxylate (**22**, 151 mg, 0.5 mmol) and NH_3_/MeOH (20%, 5 mL) were placed and stirred in a pressurised reaction vial. After a reaction of 4 h, the solvent was evaporated, and the residue was crystallised with Et_2_O (10 mL). Yield B: 126 mg (88%) M.p. 143–146 °C.

^1^H NMR (DMSO-*d*_6_); 2.38–2.49 (4H, m); 3.48–3.58 (4H, m); 3.61 (2H, s); 7.34 (1H, t, *J* = 6.8 Hz); 7.66 (1H, t, *J* = 7.3 Hz); 7.87 (1H, d, *J* = 8.2 Hz); 8.09 (1H, d, *J* = 7.2 Hz); 8.15 (1H, br ~s); 10.02 (1H, br ~s); 11.73 (1H, br ~s); ^13^C NMR (DMSO-*d*_6_); 51.3; 52.7; 67.0; 114.0; 119.9; 124.5; 124.8; 126.1; 133.0; 140.0; 144.9; 165.4; 178.1; (Figures S50 and S51).

## 5. Patents

[P1] “Novel types of C-3 substituted kinurenic acid derivatives with improved neuroprotective activity”. Patent application PCT/HU2017/000014, September 2017.

## Figures and Tables

**Table 1:** Diagnostic ^1^H- and ^13^C chemical shifts measured for **19a**,**b** and calculated for the corresponding oxo- and enol forms by GIAO method ^a^.

	19a			19b		
	Experimental ^1^H- and ^13^C NMR Chemical Shift	oxo Calculated ^1^H- and ^13^C NMR Chemical Shift	enol Calculated ^1^H- and ^13^C NMR Chemical Shift	Experimental ^1^H- and ^13^C NMR Chemical Shift	oxo Calculated ^1^H- and ^13^C NMR Chemical Shift	enol Calculated ^1^H- and ^13^C NMR Chemical Shift
XH b	11.70	9.60	15.05	11.70	9.64	16.51
10-NH	11.31	12.78	8.88	11.32	12.72	8.22
H-5	8.05	8.61	8.73	8.03	8.62	8.65
H-6	7.26	7.72	7.98	7.28	7.72	7.96
H-7	7.58	8.07	8.16	7.60	8.07	8.09
H-8	7.81	7.83	8.37	7.84	7.82	8.30
C-2	143.5	148.3	153.7	143.9	148.2	156.2
C-3	115.1	124.3	119.5	114.2	124.2	118.8
C-4	177.3	186.3	173.5	177.8	186.2	170.0
C-4a	119.6	131.7	127.8	119.8	131.4	127.7
C-5	125.6	130.2	128.0	125.6	130.2	128.5
C-6	123.9	129.7	132.2	124.1	129.8	132.5
C-7	132.5	138.8	136.9	132.6	138.8	136.5
C-8	119.6	123.5	135.0	119.7	123.5	135.5
C-8a	139.6	144.2	152.6	139.7	144.2	153.7
C-10	163.3	171.2	173.4	163.5	171.4	174.4
C-18	47.5	49.6	58.2	50.8	53.5	62.8

^a^ Calculations were performed using B3LYP functional and 6-311++G(2d,p) basis set. ^b^ NH in the oxo tautomers, OH in the enol tautomers.

## Supplementary Materials

The following are available online, Figures S1–S53: NMR spectra of synthesized compounds.
