# Supplementary material for: Synthesis of New C-3 Substituted Kynurenic Acid Derivatives
Source: Molecules. 2020 Feb 19;25(4):937. doi: 10.3390/molecules25040937 (PMC7071119; doi:10.3390/molecules25040937)
Supplement: Supplementary file 1 [file molecules-25-00937-s001.pdf]

Supplementary Information

Synthesis of New C-3 Substituted Kynurenic Acid Derivatives

Bálint Lőrinczi [1,2], Antal Csámpai [3], Ferenc Fülöp [1,2] and István Szatmári [1,2,*]

[1] Institute of Pharmaceutical Chemistry and Research Group for Stereochemistry, Hungarian Academy of Sciences, University of Szeged, H-6720 Szeged, Eötvös u. 6, Hungary; lorinczi.balint@pharm.u-szeged.hu (B.L.); fulop@pharm.u-szeged.hu (F.F.)

[2] Institute of Pharmaceutical Chemistry, University of Szeged, Interdisciplinary Excellence Center; H-6720 Szeged, Eötvös u. 6, Hungary

[3] Department of Inorganic Chemistry, Eötvös Loránd University (ELTE), Pázmány P. sétány 1/A, H-1117 Budapest, Hungary; csampai@caesar.elte.hu

* Correspondence: szatmari.istvan@pharm.u-szeged.hu; Tel.: +36-62-341-966

3-(((2-Morpholinoethyl)amino)methyl)-4-oxo-1,4-dihydroquinoline-2-carboxylic acid (2)

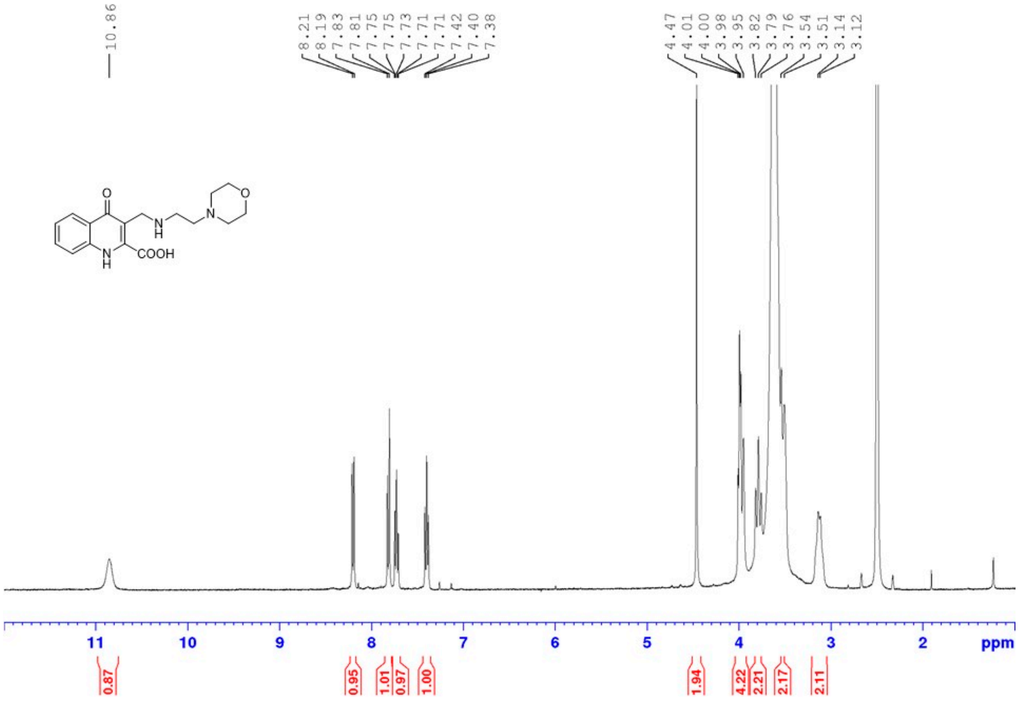

Figure S1. ^{1}H-NMR spectrum of **2**

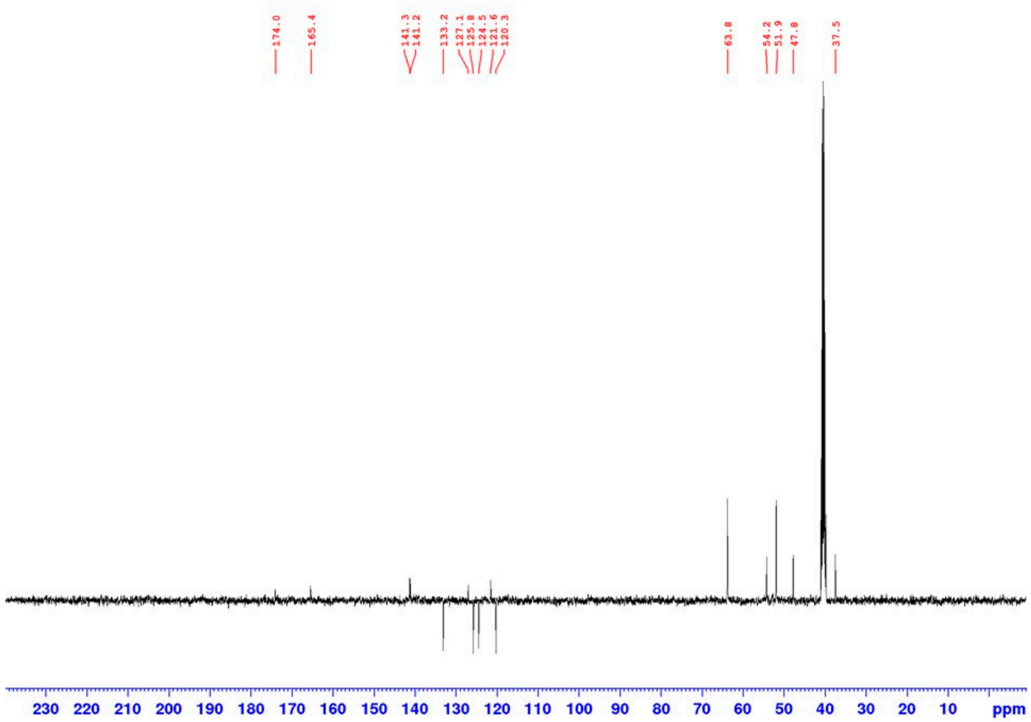

Figure S2. ^{13}C-NMR spectrum of **2**

3-(((2-(Dimethylamino)ethyl)amino)(phenyl)methyl)-4-oxo-1,4-dihydroquinoline-2-carboxylic acid (3)

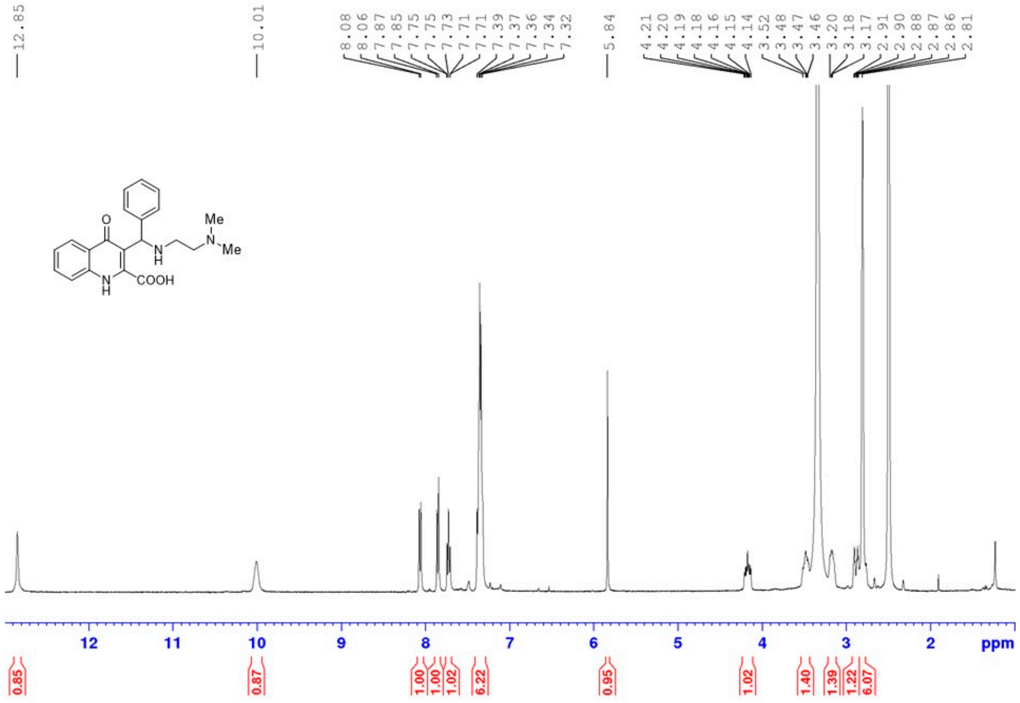

Figure S3. ^{1}H-NMR spectrum of **3**

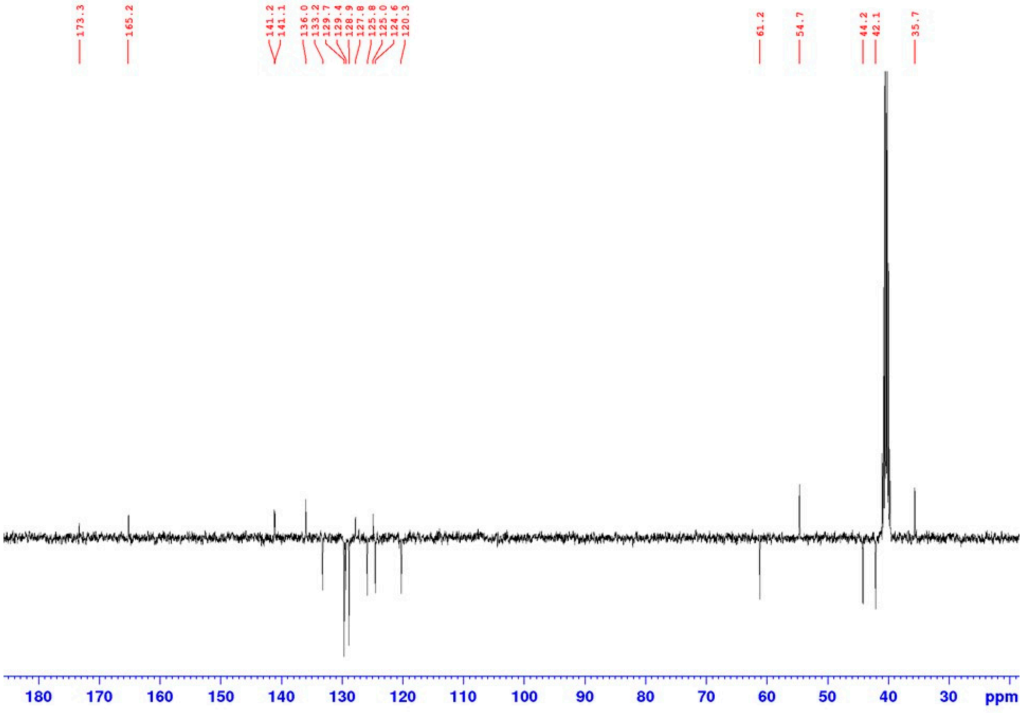

Figure S4. ^{13}C-NMR spectrum of **3**

3-((Dimethylamino)methyl)-4-oxo-1,4-dihydroquinoline-2-carboxylic acid (4a)

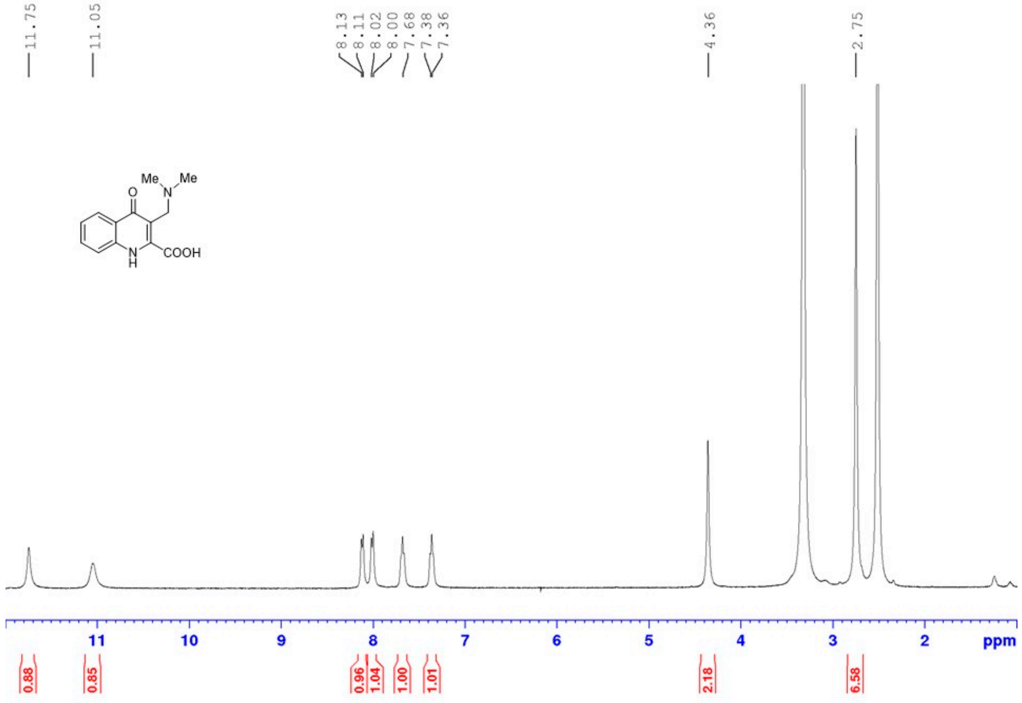

Figure S5. ^{1}H-NMR spectrum of **4a**

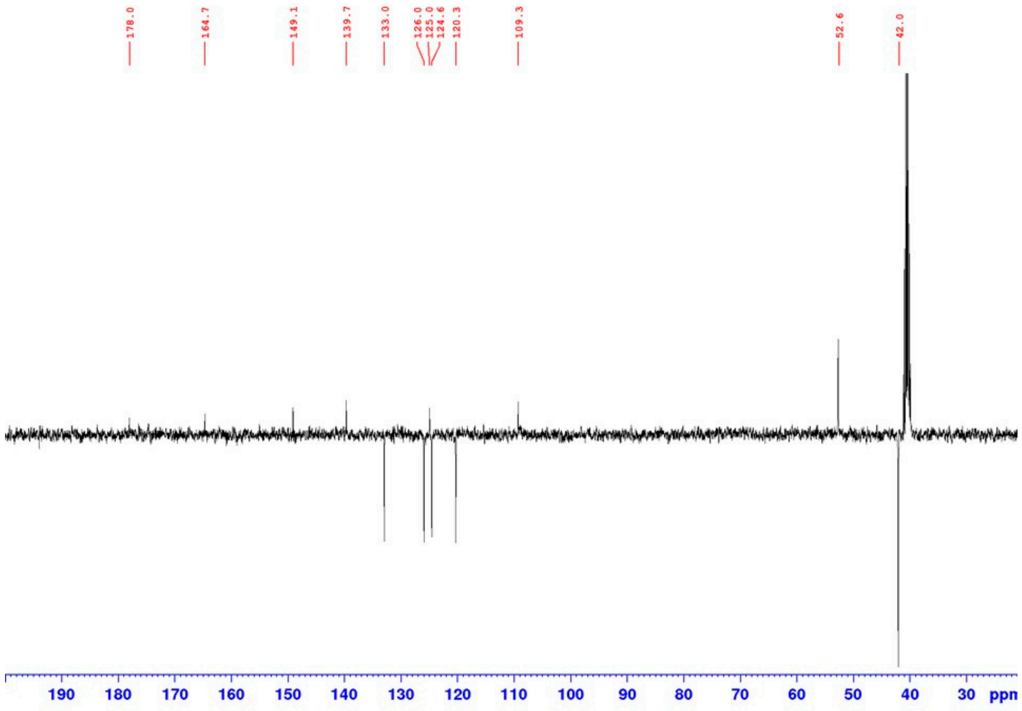

Figure S6. ^{13}C-NMR spectrum of **4a**

3-((Benzyl(methyl)amino)methyl)-4-oxo-1,4-dihydroquinoline-2-carboxylic acid (4b)

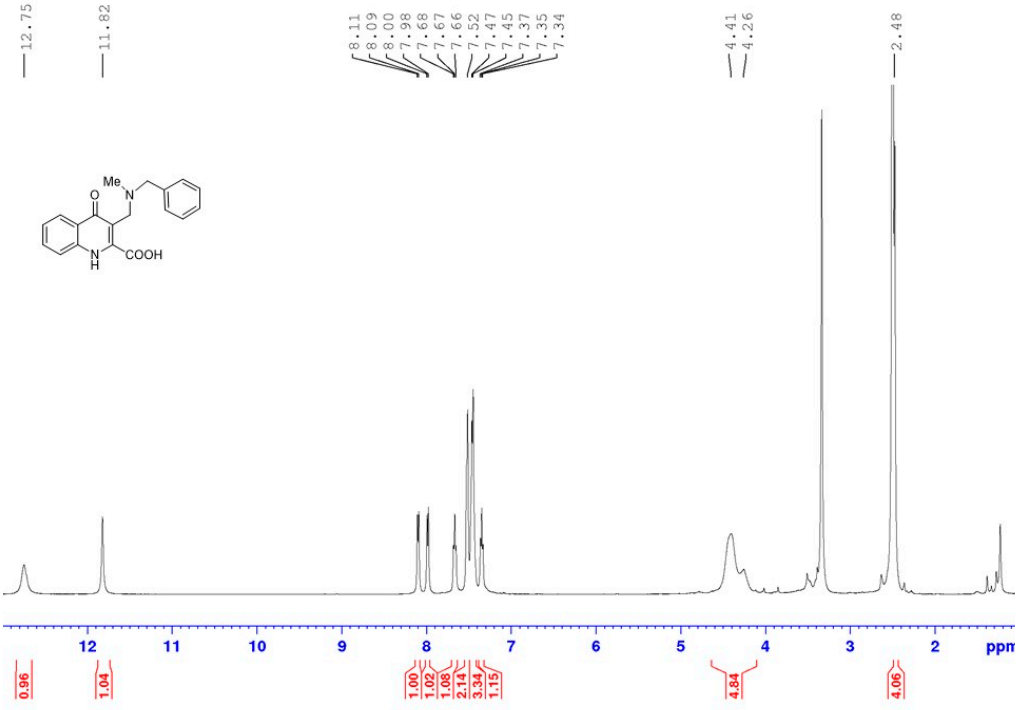

Figure S7. ^{1}H-NMR spectrum of **4b**

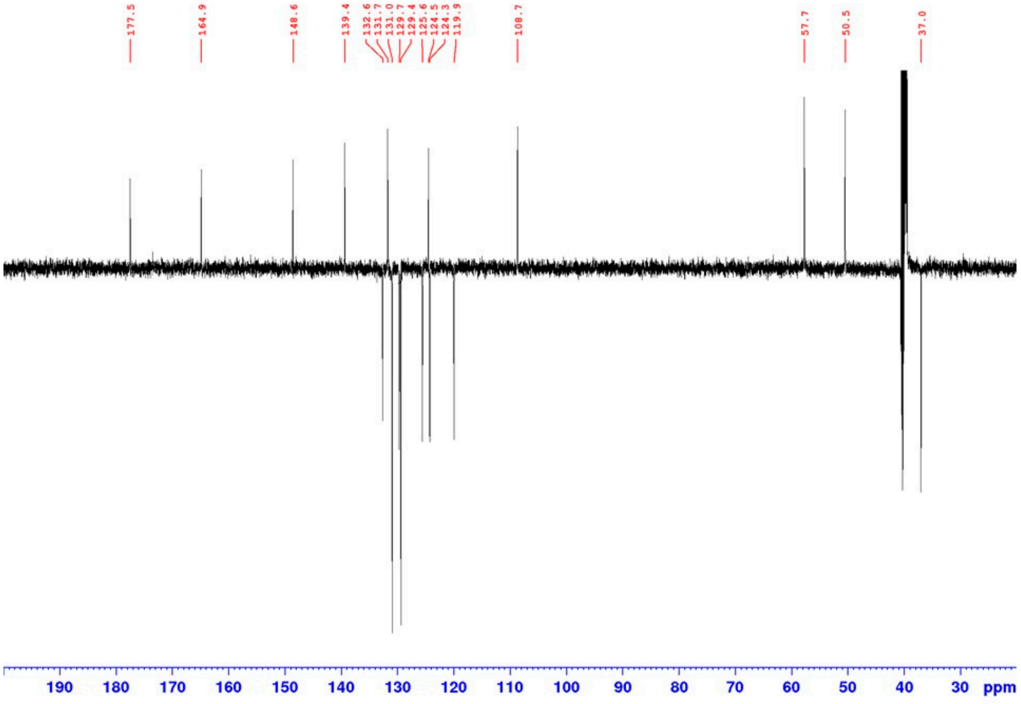

Figure S8. ^{13}C-NMR spectrum of **4b**

4-Oxo-3-(piperidin-1-ylmethyl)-1,4-dihydroquinoline-2-carboxylic acid (5b)

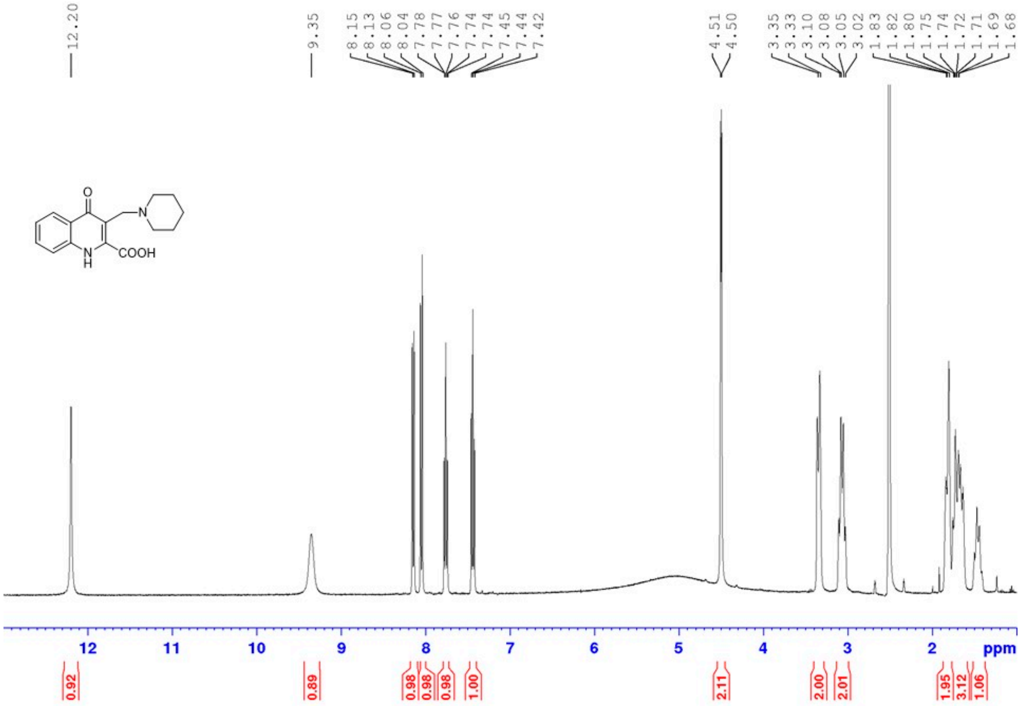

Figure S9. ^{1}H-NMR spectrum of **5b**

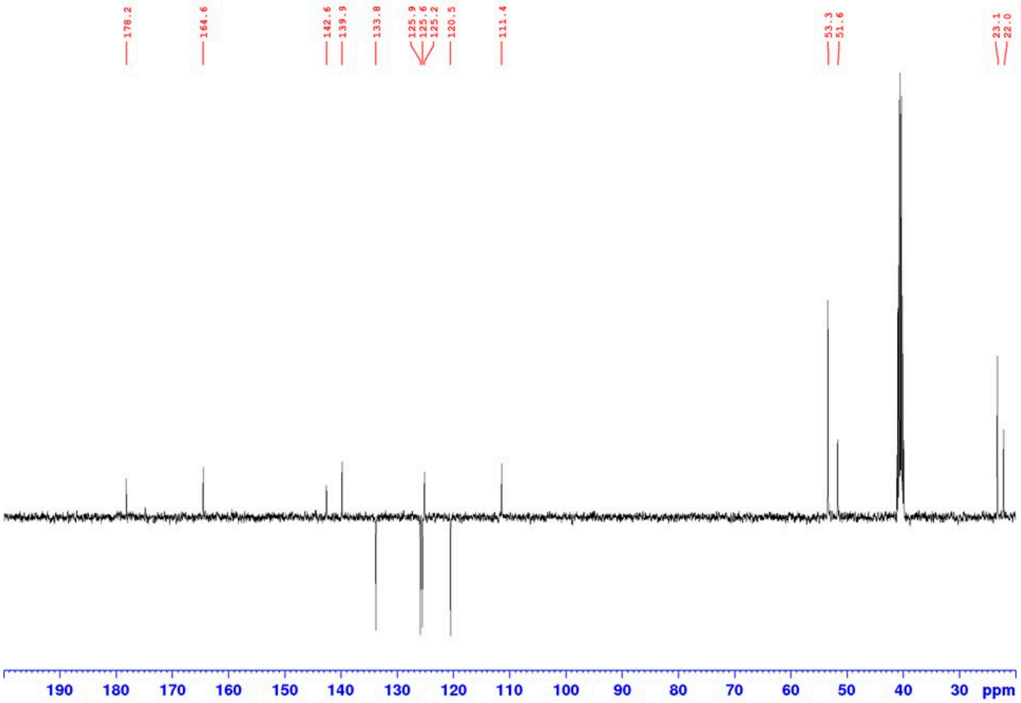

Figure S10. ^{13}C-NMR spectrum of **5b**

3-((4-Methylpiperazin-1-yl)methyl)-4-oxo-1,4-dihydroquinoline-2-carboxylic acid (5c)

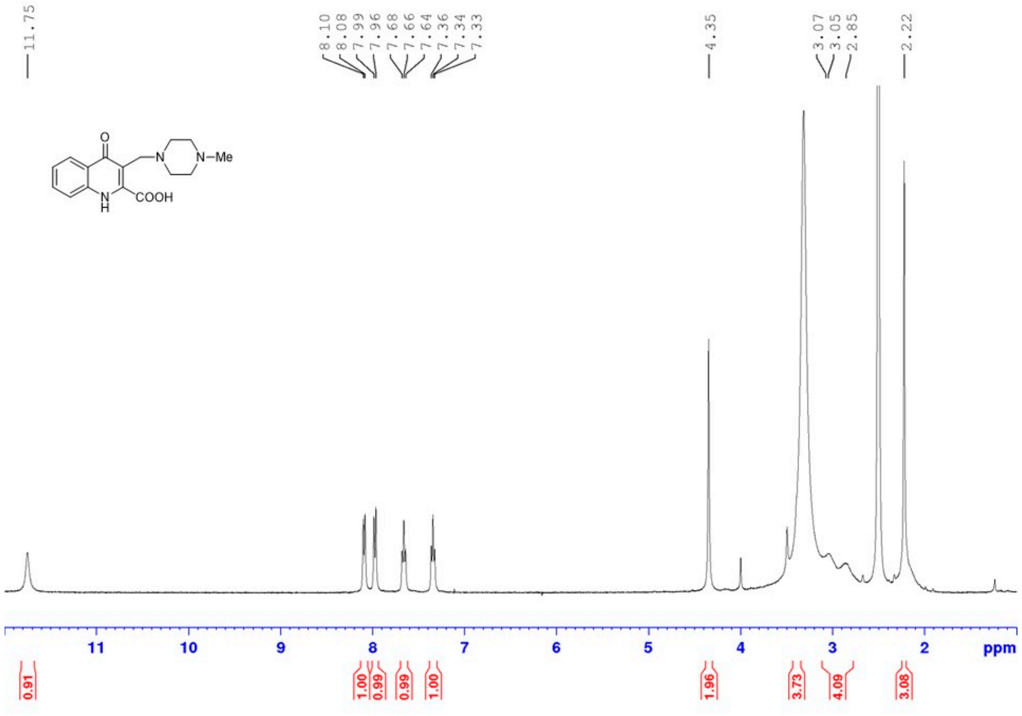

Figure S11. ^{1}H-NMR spectrum of **5c**

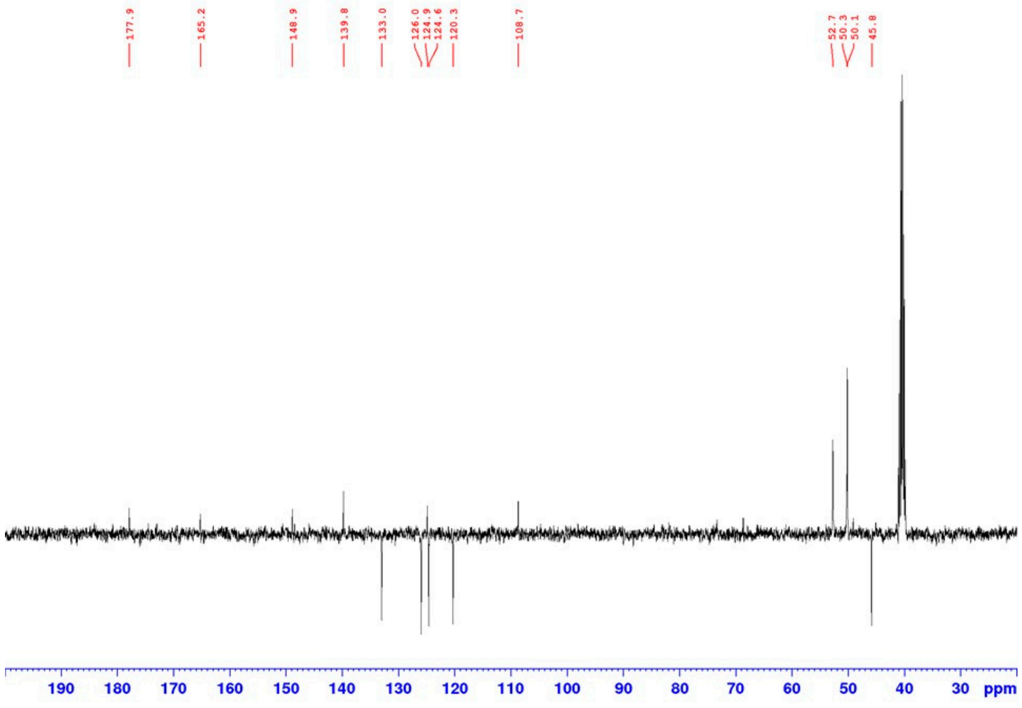

Figure S12. ^{13}C-NMR spectrum of **5c**

3-((3,4-Dihydroisoquinolin-2(1H)-yl)methyl)-4-oxo-1,4-dihydroquinoline-2-carboxylic acid (6a)

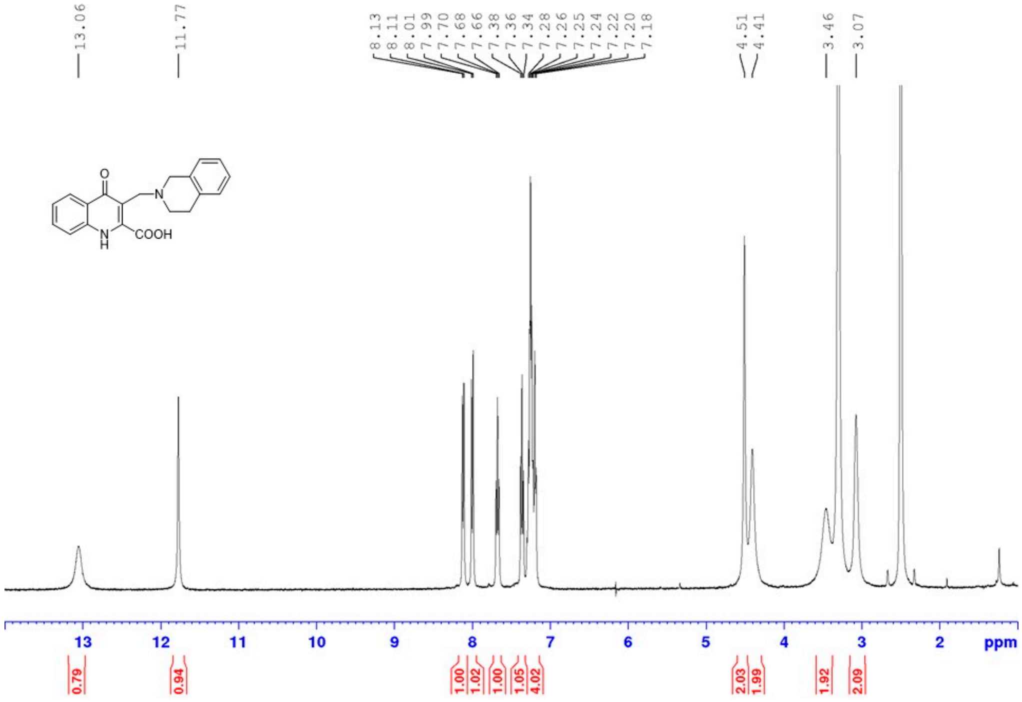

Figure S13. ^{1}H-NMR spectrum of **6a**

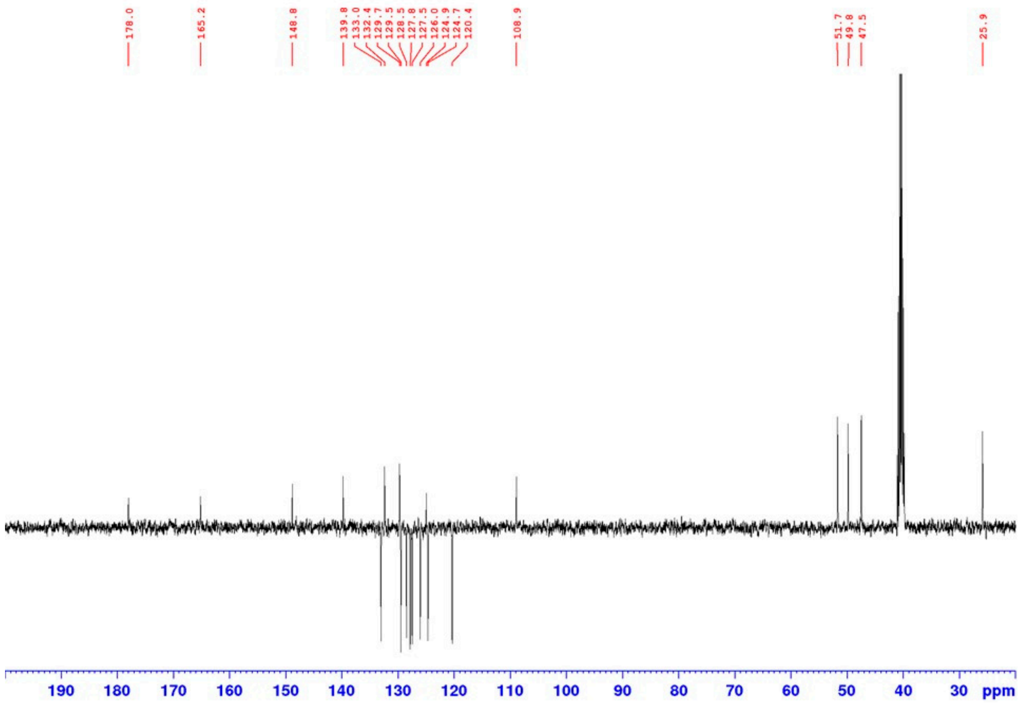

Figure S14. ^{13}C-NMR spectrum of **6a**

3-((6,7-Dimethoxy-3,4-dihydroisoquinolin-2(1H)-yl)methyl)-4-oxo-1,4-dihydroquinoline-2-carboxylic acid (6b)

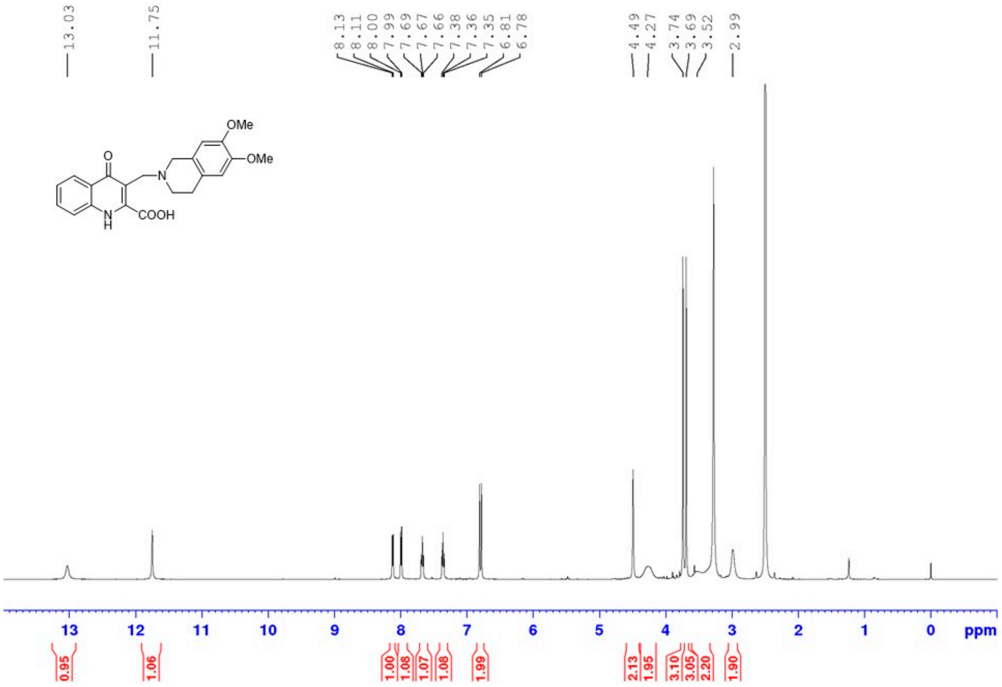

Figure S15. ^{1}H-NMR spectrum of **6b**

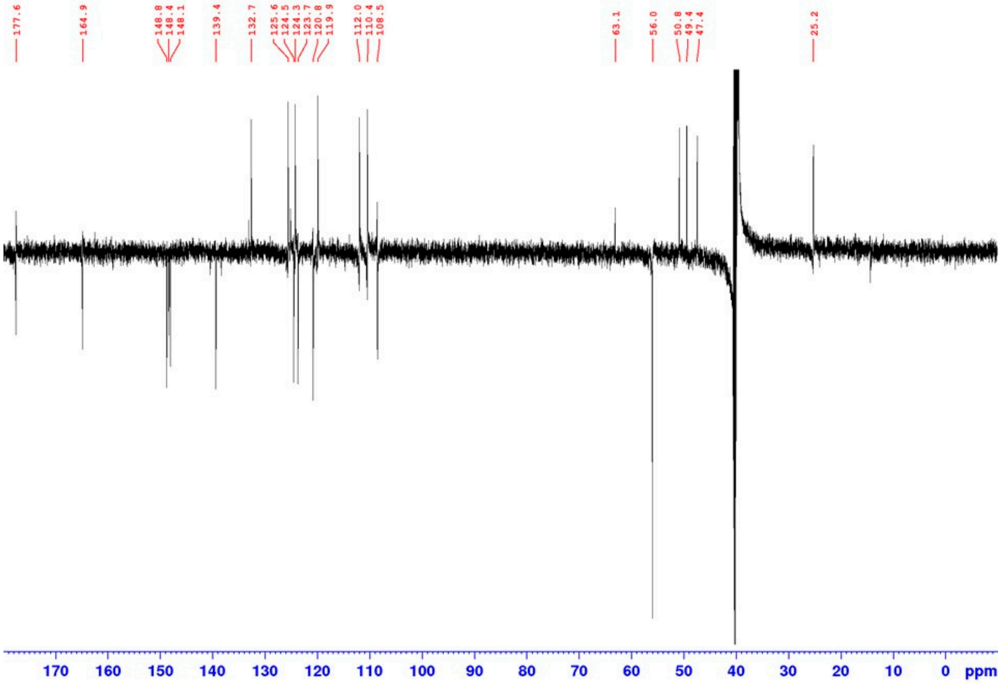

Figure S16. ^{13}C-NMR spectrum of **6b**

Ethyl 5-chloro-4-oxo-1,4-dihydroquinoline-2-carboxylate (7)

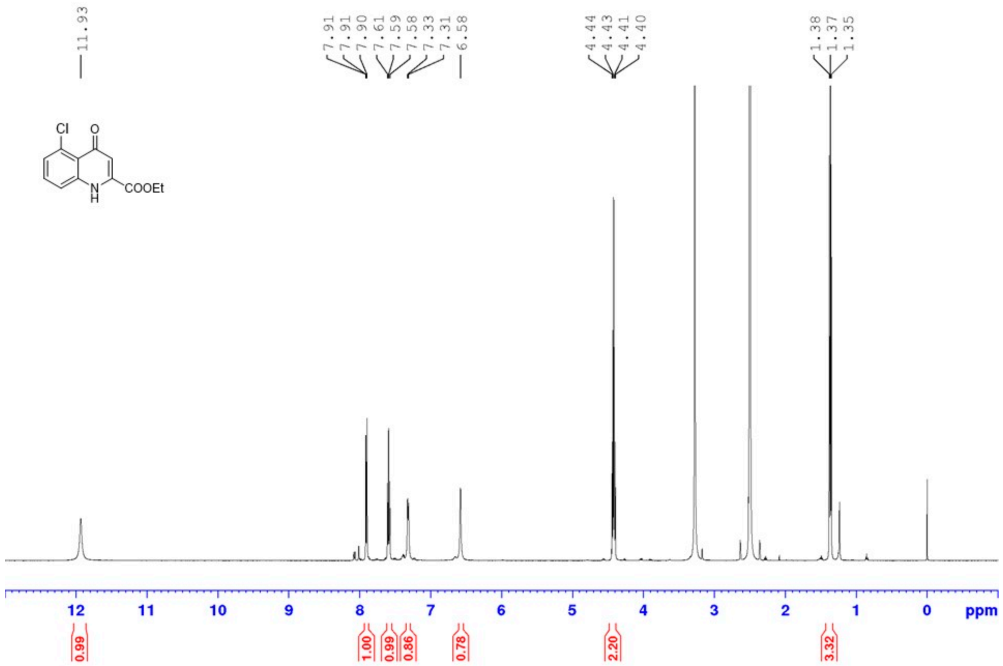

Figure S17. ^{1}H-NMR spectrum of **7**

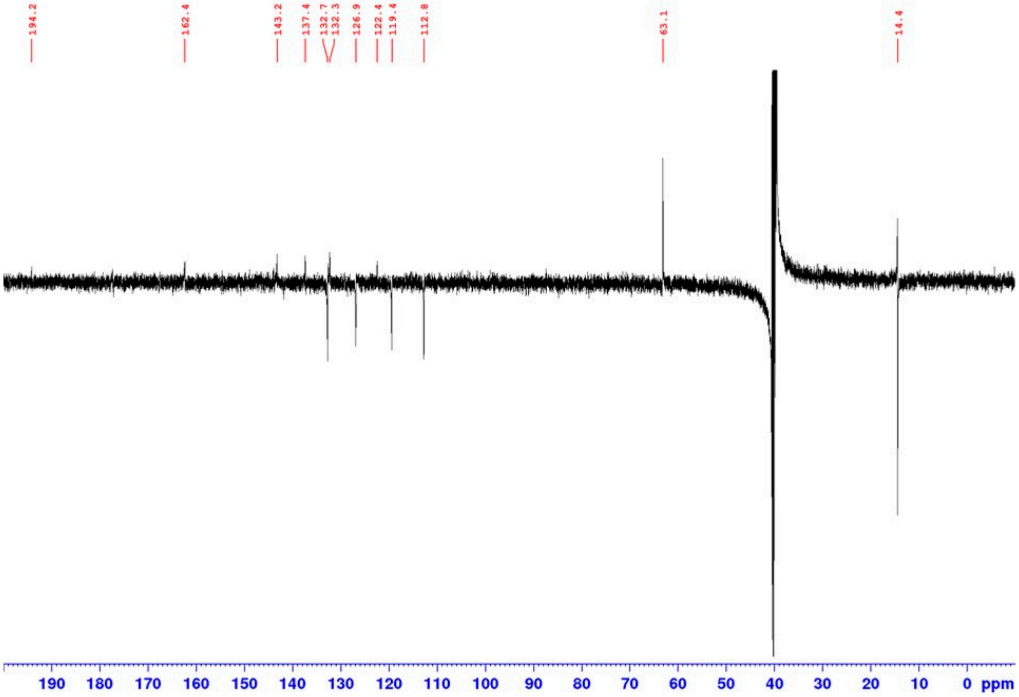

Figure S18. ^{13}C-NMR spectrum of **7**

Ethyl 4-oxo-6-phenyl-1,4-dihydroquinoline-2-carboxylate (8)

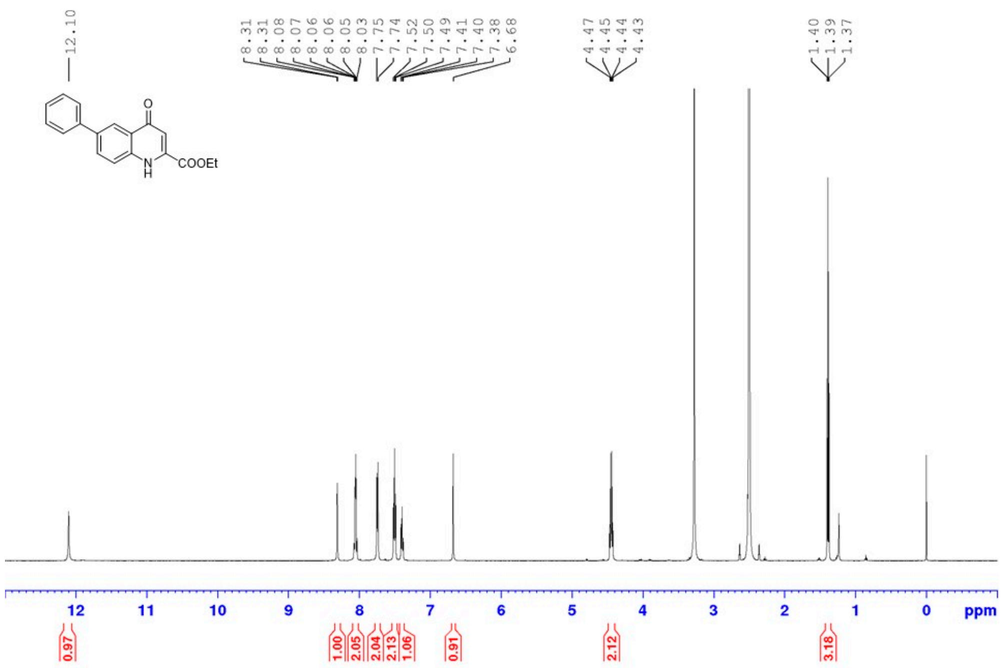

Figure S19. ^{1}H-NMR spectrum of **8**

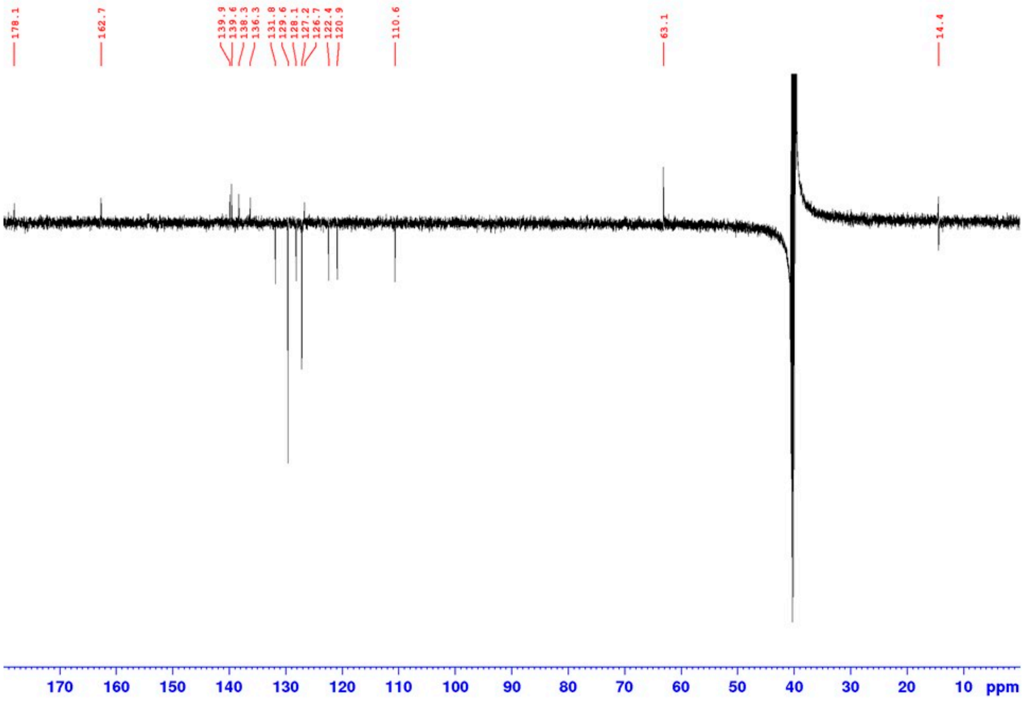

Figure S20. ^{13}C-NMR spectrum of **8**

Ethyl 7-chloro-4-oxo-1,4-dihydroquinoline-2-carboxylate (9)

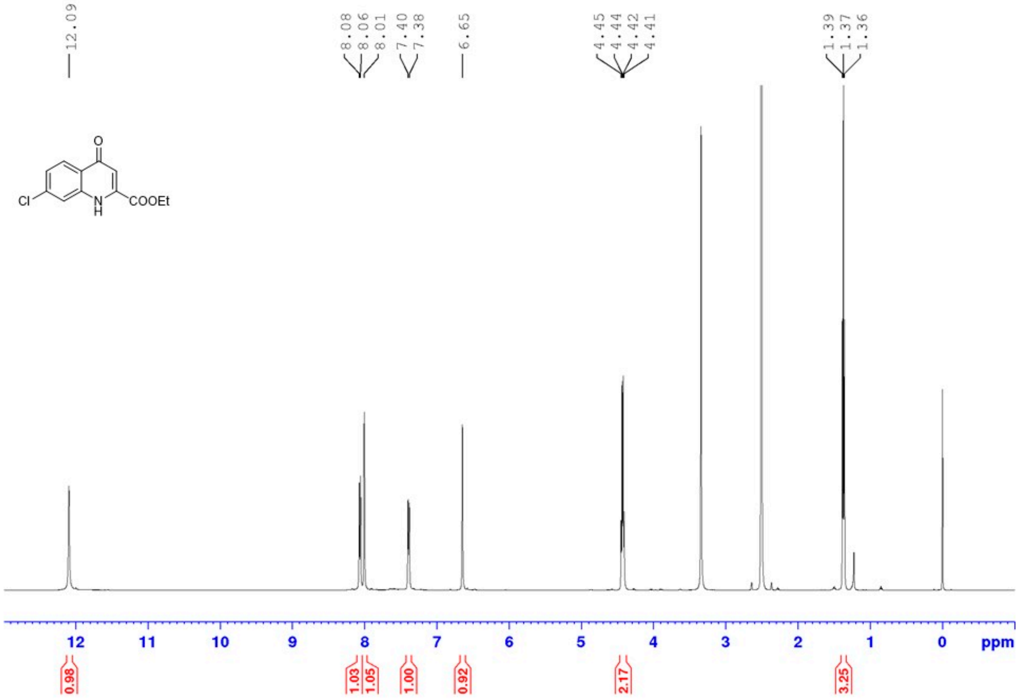

Figure S21. ^{1}H-NMR spectrum of **9**

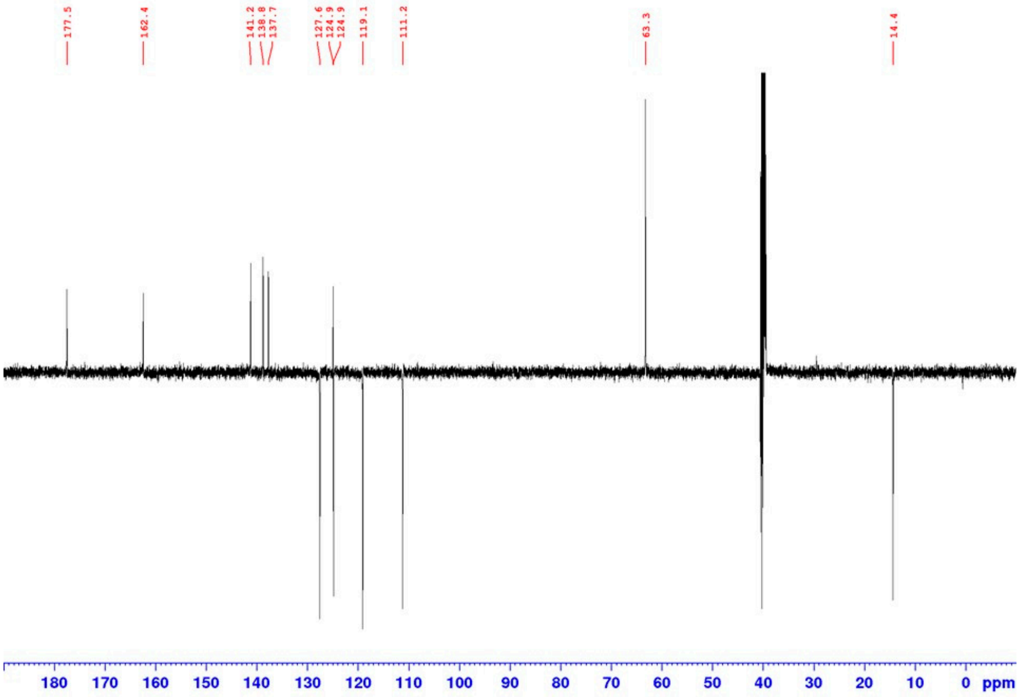

Figure S22. ^{13}C-NMR spectrum of **9**

Ethyl 4-oxo-8-methyl-1,4-dihydroquinoline-2-carboxylate (10)

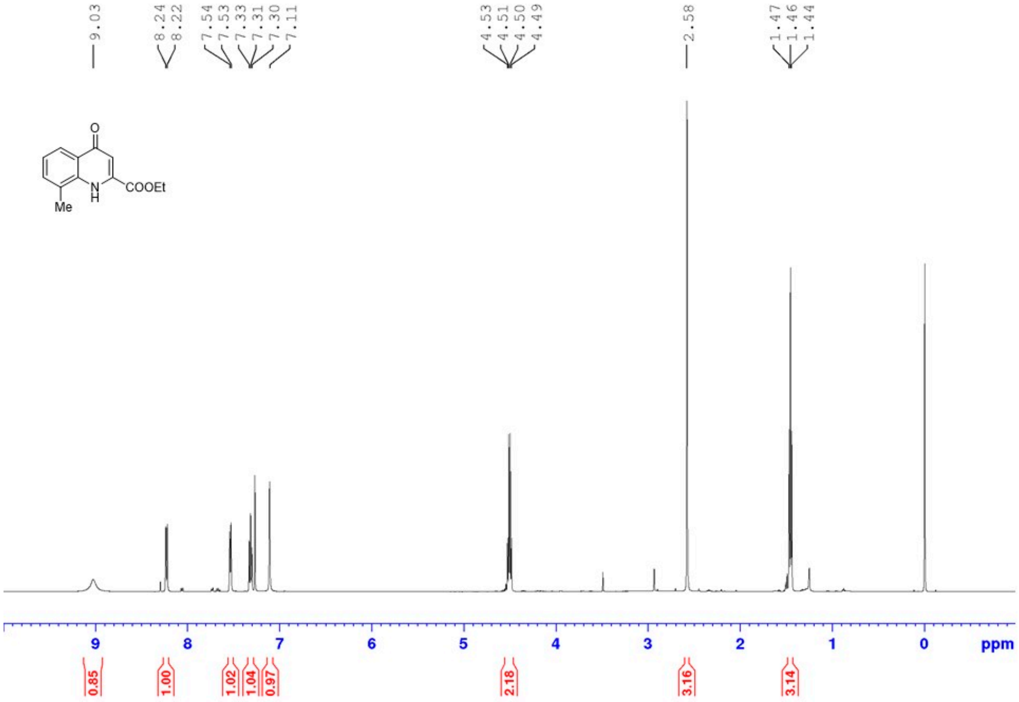

Figure S23. ^{1}H-NMR spectrum of **10**

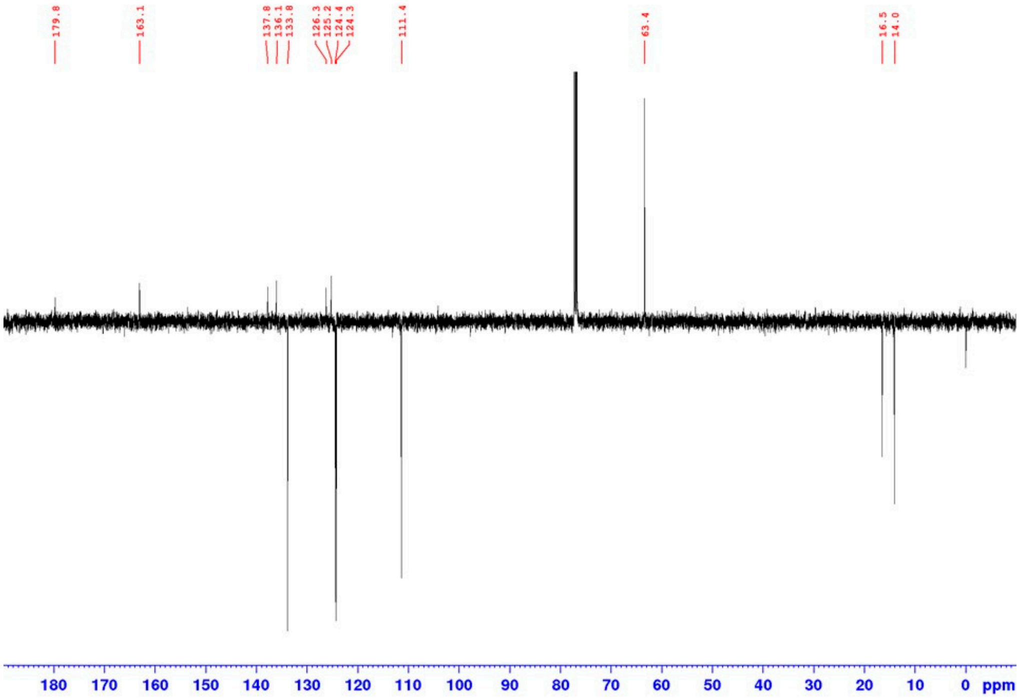

Figure S24. ^{13}C-NMR spectrum of **10**

5-Chloro-3-(morpholinomethyl)-4-oxo-1,4-dihydroquinoline-2-carboxylic acid (11)

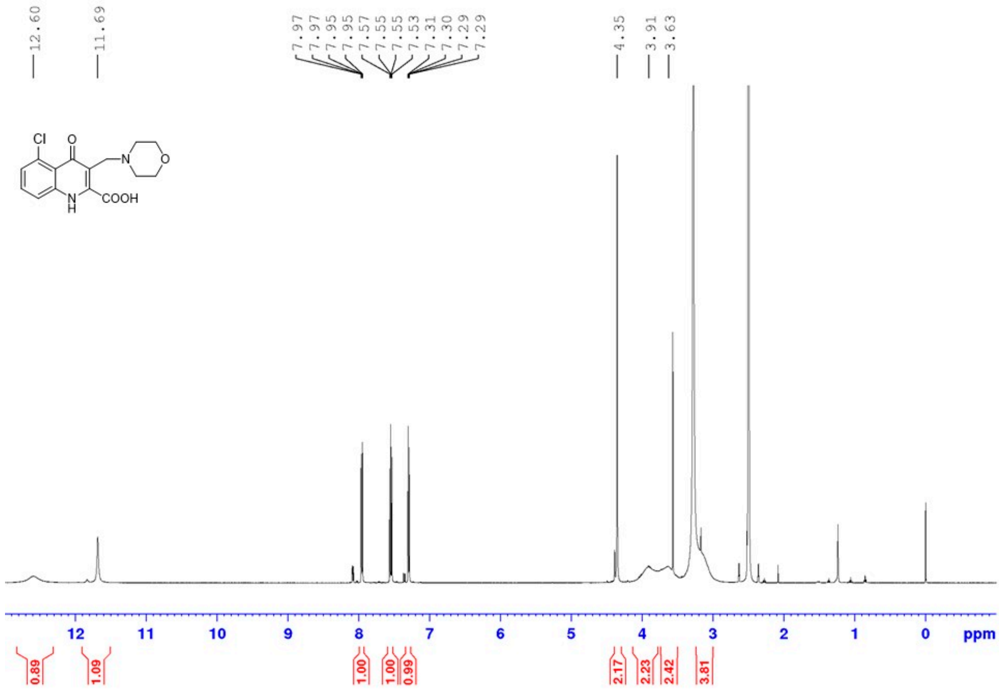

Figure S25. ^{1}H-NMR spectrum of **11**

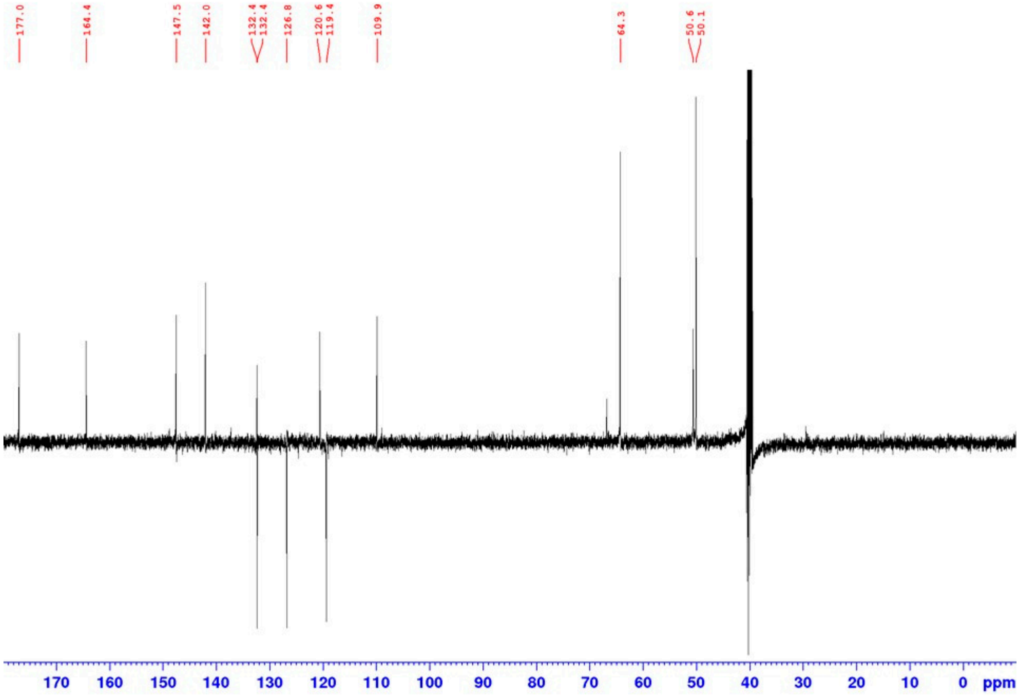

Figure S26. ^{13}C-NMR spectrum of **11**

3-(Morpholinomethyl)-4-oxo-6-phenyl-1,4-dihydroquinoline-2-carboxylic acid (12)

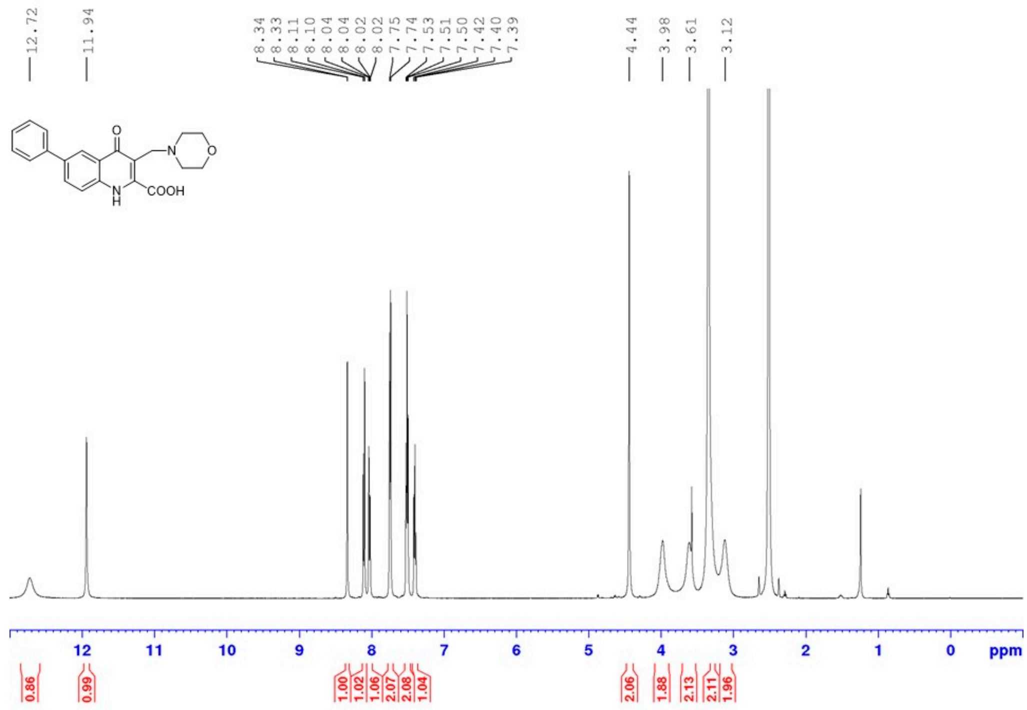

Figure S27. ^{1}H-NMR spectrum of **12**

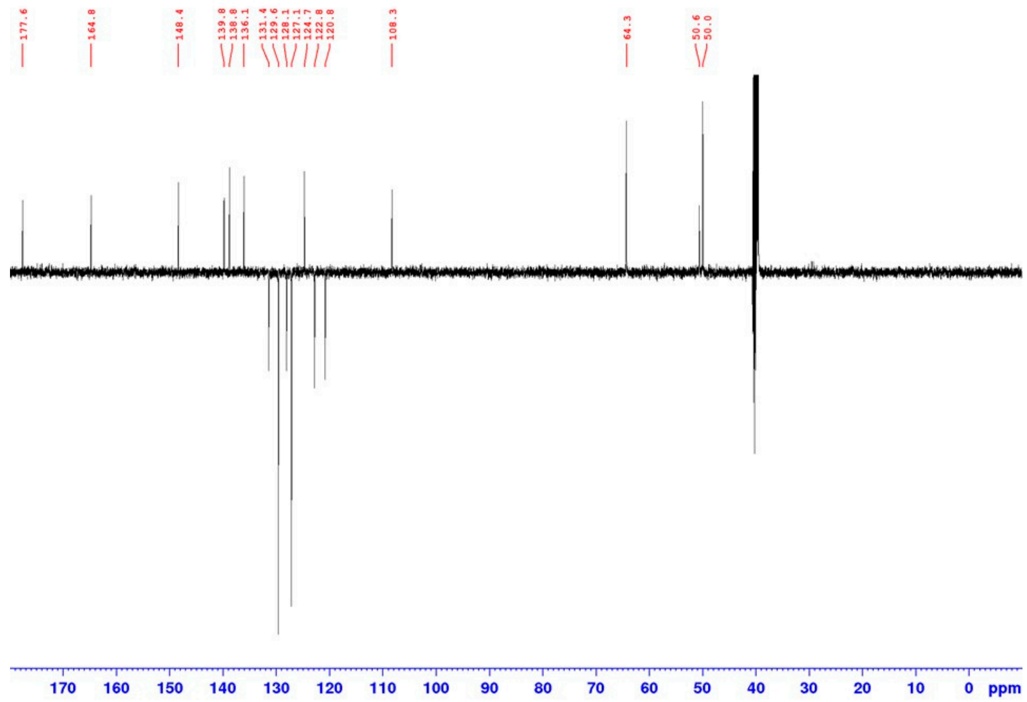

Figure S28. ^{13}C-NMR spectrum of **12**

7-Chloro-3-(morpholinomethyl)-4-oxo-1,4-dihydroquinoline-2-carboxylic acid (13)

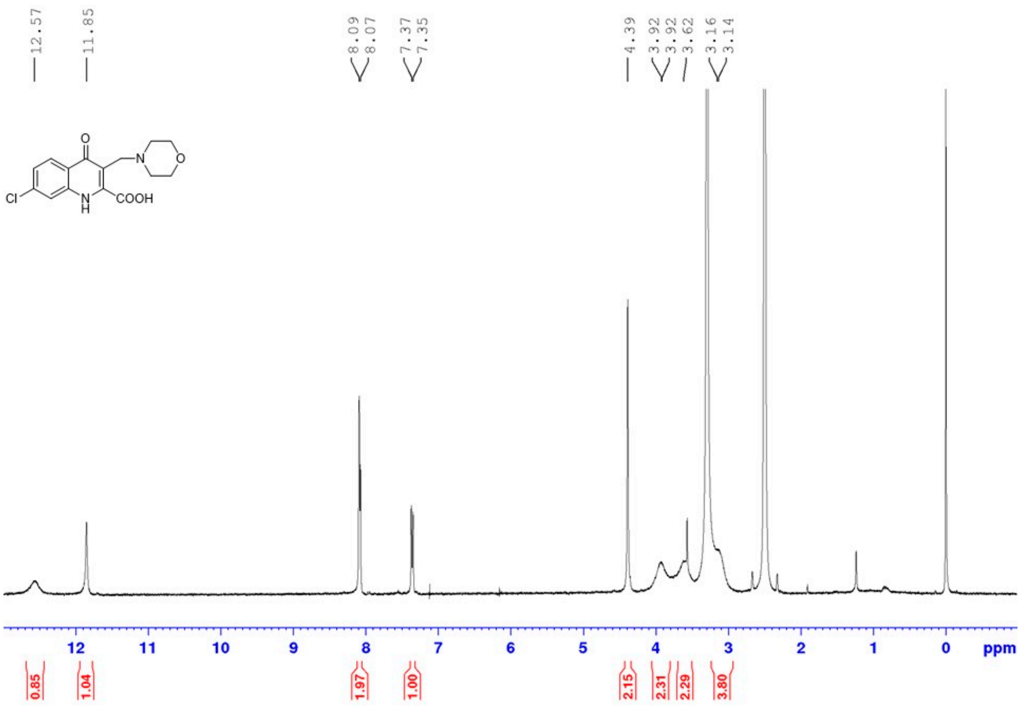

Figure S29. ^{1}H-NMR spectrum of **13**

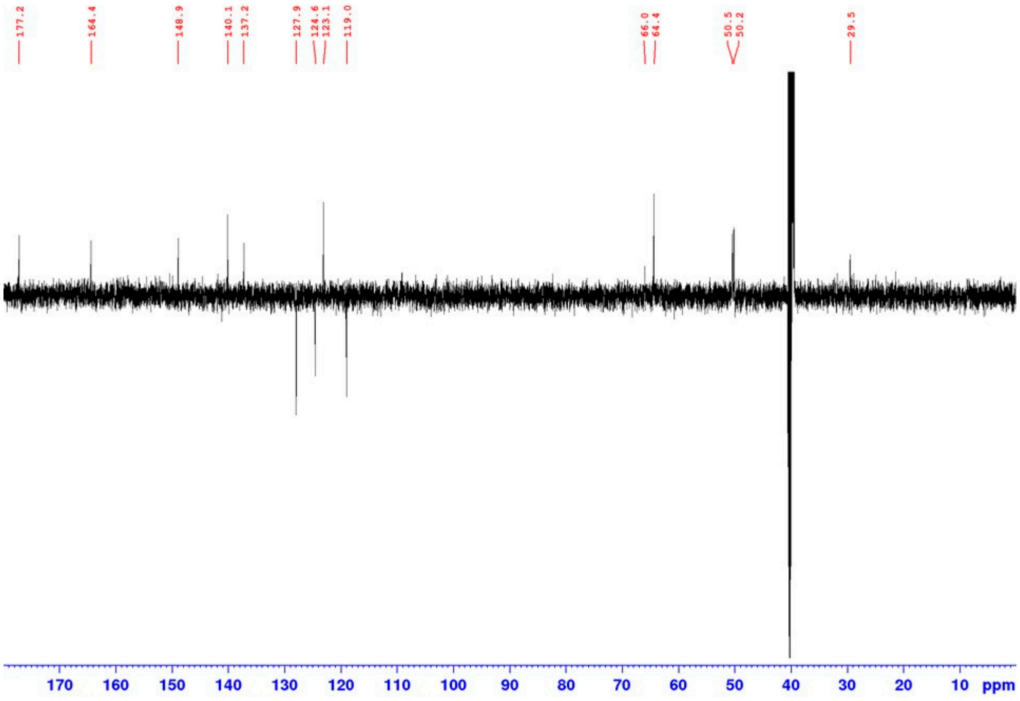

Figure S30. ^{13}C-NMR spectrum of **13**

8-Methyl-3-(morpholinomethyl)-4-oxo-1,4-dihydroquinoline-2-carboxylic acid (14)

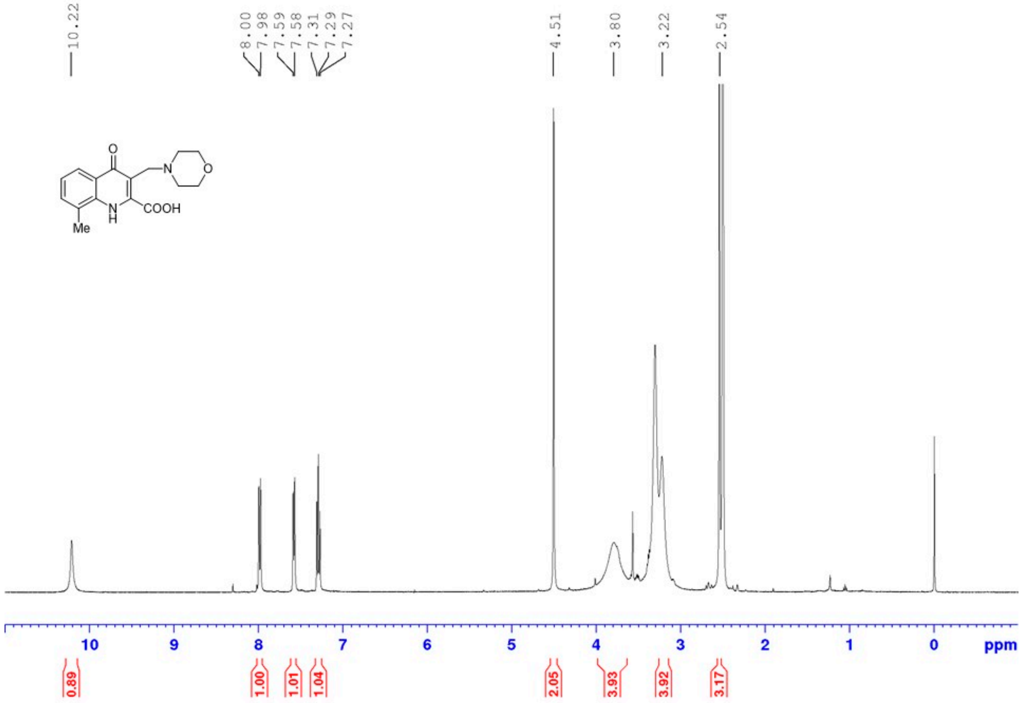

Figure S31. ^{1}H-NMR spectrum of **14**

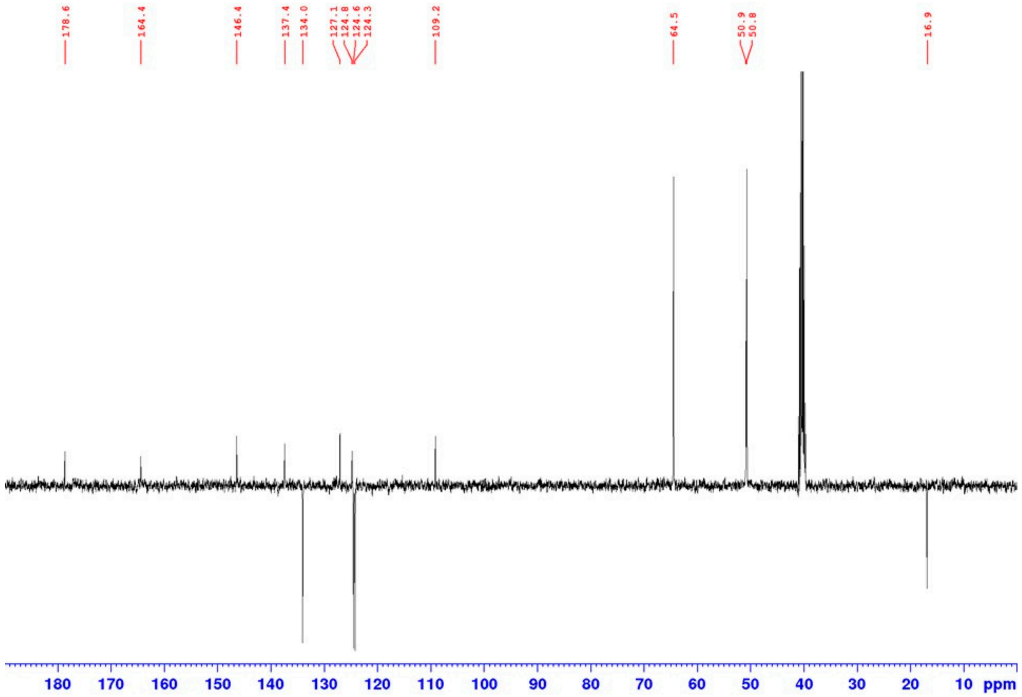

Figure S32. ^{13}C-NMR spectrum of **14**

***N*-(2-(Dimethylamino)ethyl)-4-oxo-3-(pyrrolidin-1-ylmethyl)-1,4-dihydroquinoline-2-carboxamide (18a)**

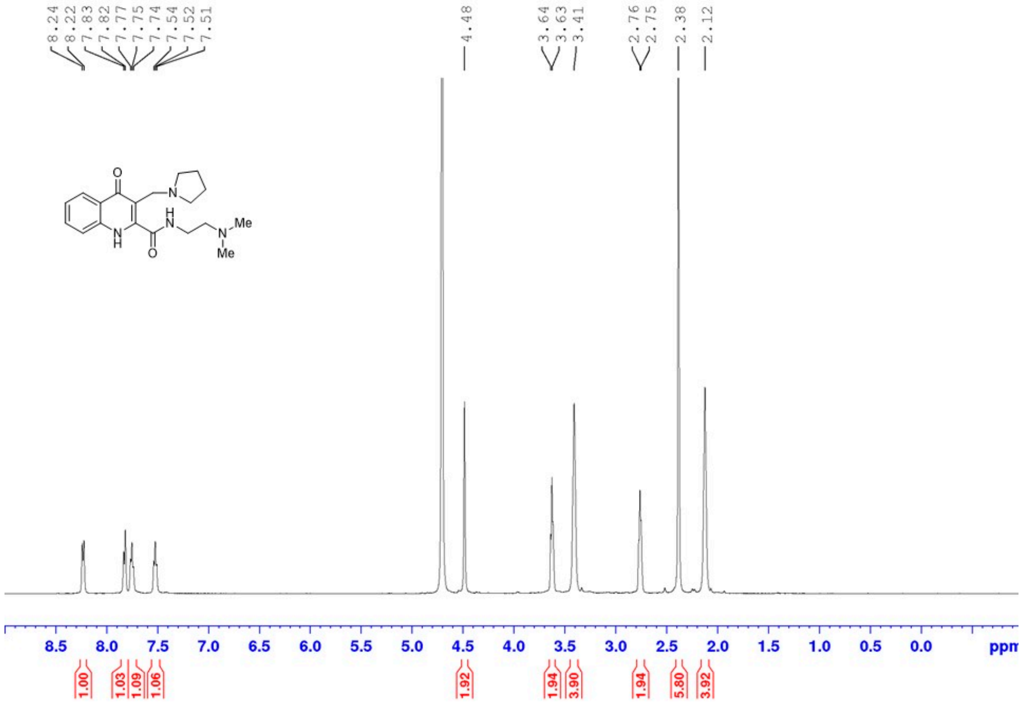

Figure S33. ^{1}H-NMR spectrum of **18a**

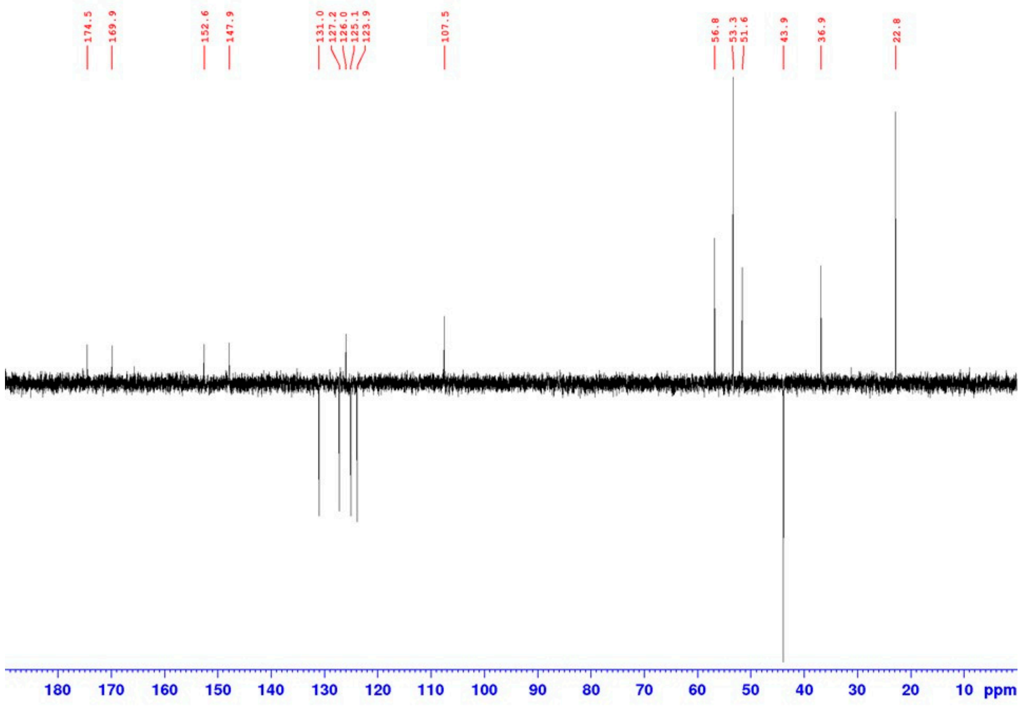

Figure S34. ^{13}C-NMR spectrum of **18a**

***N*-(2-(Dimethylamino)ethyl)-4-oxo-3-(piperidin-1-ylmethyl)-1,4-dihydroquinoline-2-carboxamide (18b)**

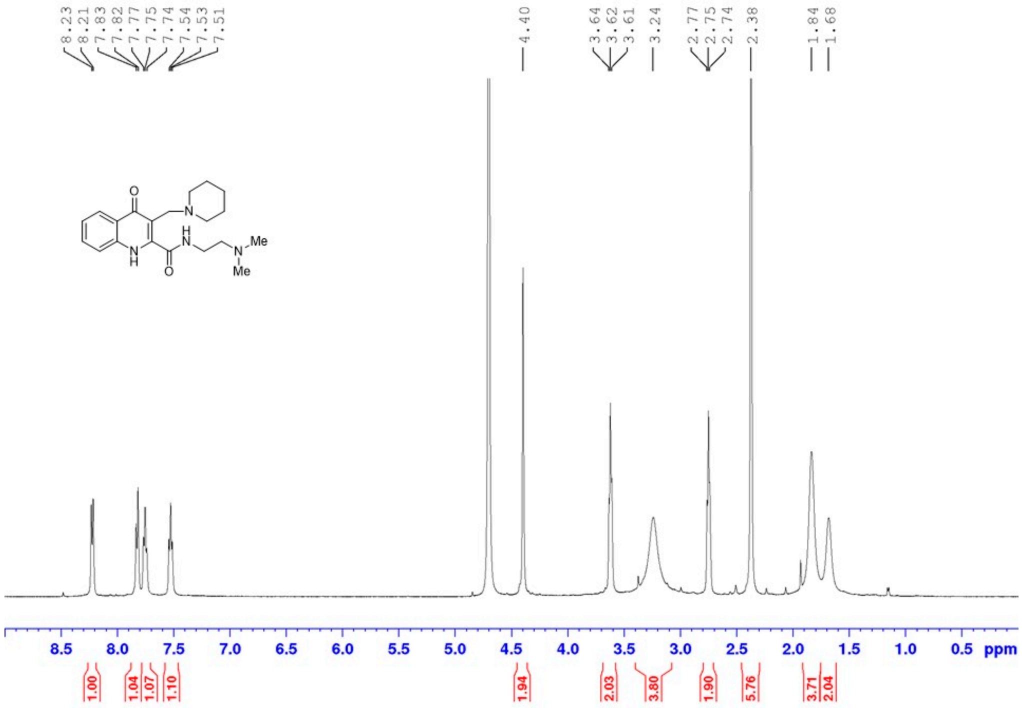

Figure S35. ¹H-NMR spectrum of **18b**

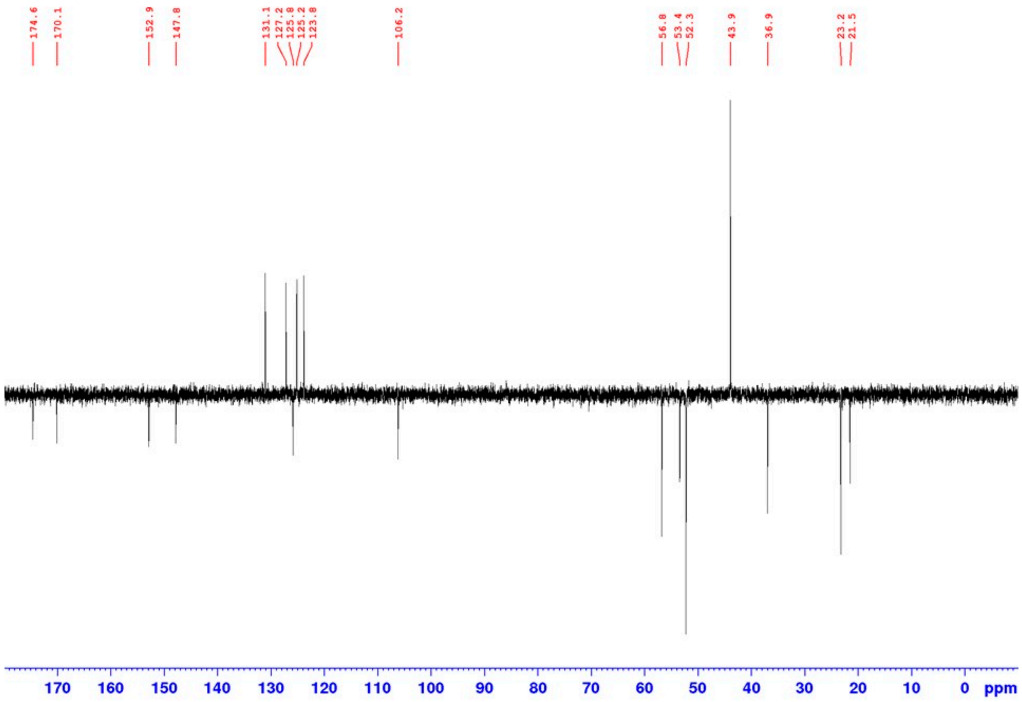

Figure S36. ¹³C-NMR spectrum of **18b**

N-(2-(Dimethylamino)ethyl)-3-(morpholinomethyl)-4-oxo-1,4-dihydroquinoline-2-carboxamide (18c)

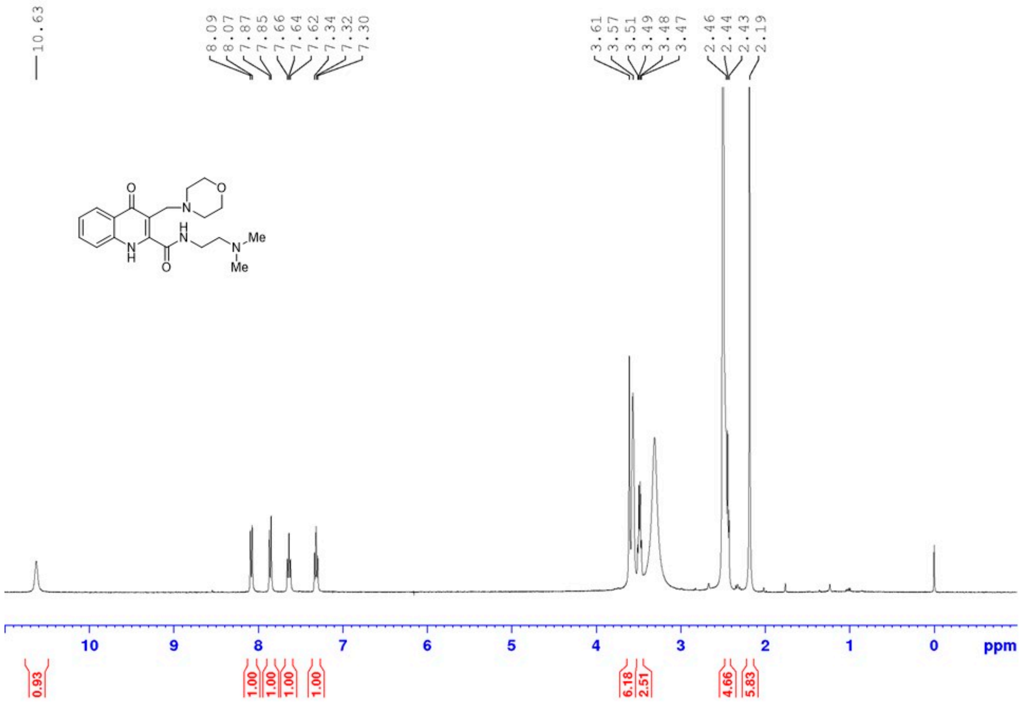

Figure S37. ^{1}H-NMR spectrum of **18c**

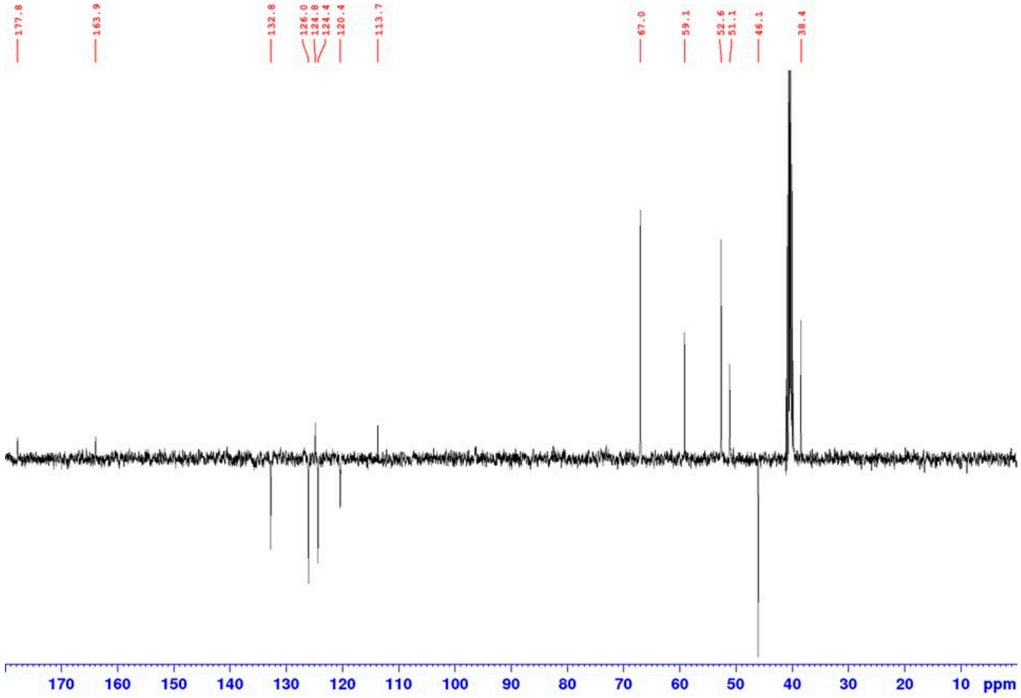

Figure S38. ^{13}C-NMR spectrum of **18c**

4-oxo-*N*-(2-(pyrrolidin-1-yl)ethyl)-3-(pyrrolidin-1-ylmethyl)-1,4-dihydroquinoline-2-carboxamide (19a)

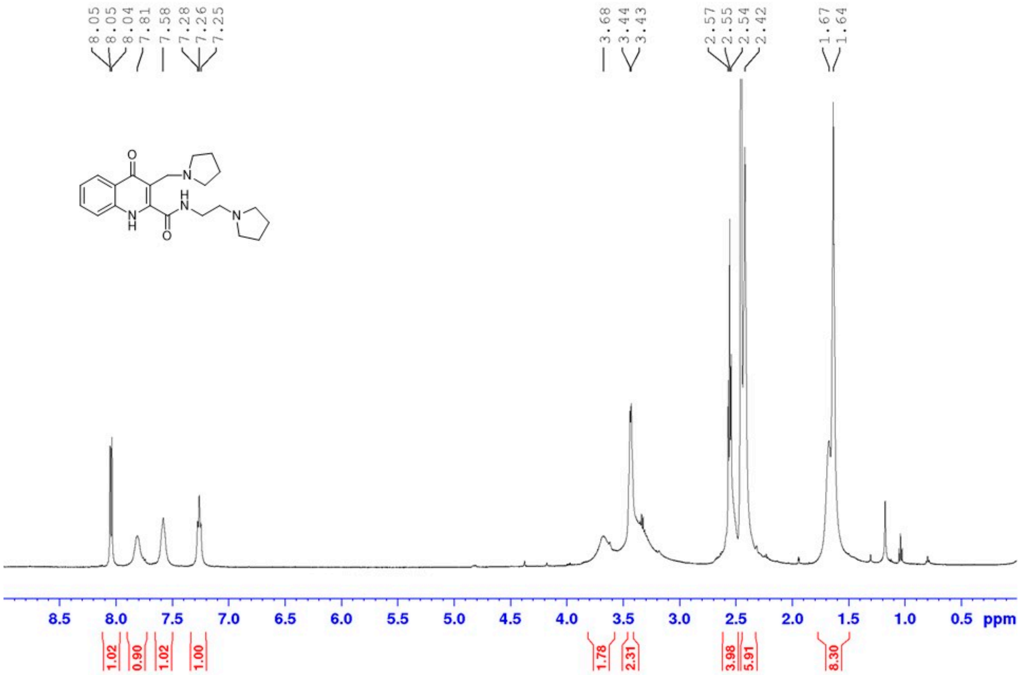

Figure S39. ^{1}H-NMR spectrum of **19a**

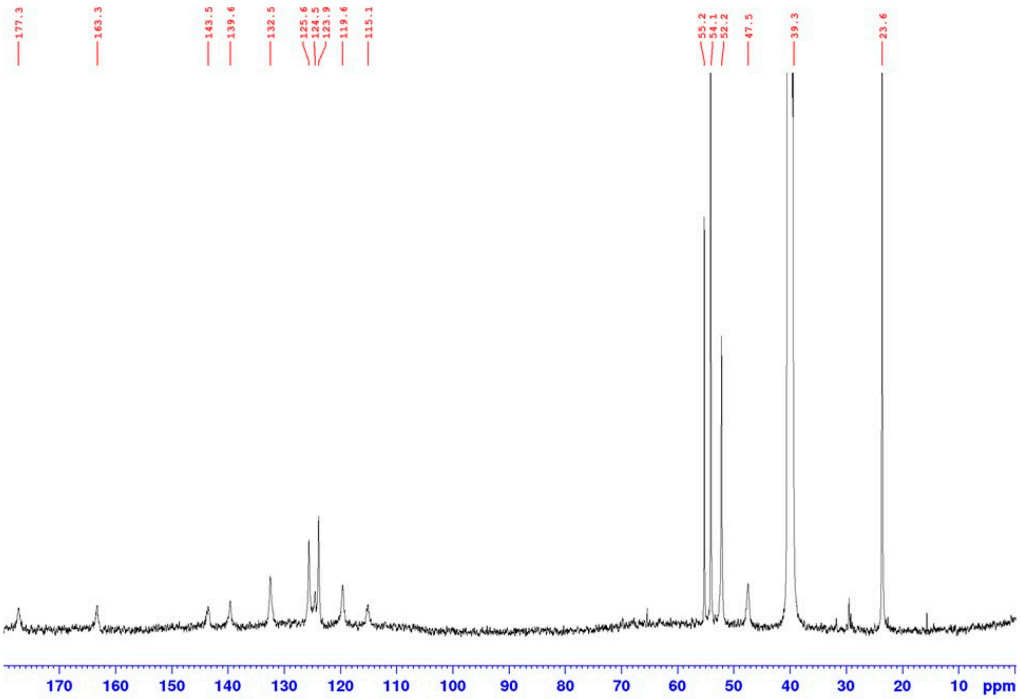

Figure S40. ^{13}C-NMR spectrum of **19a**

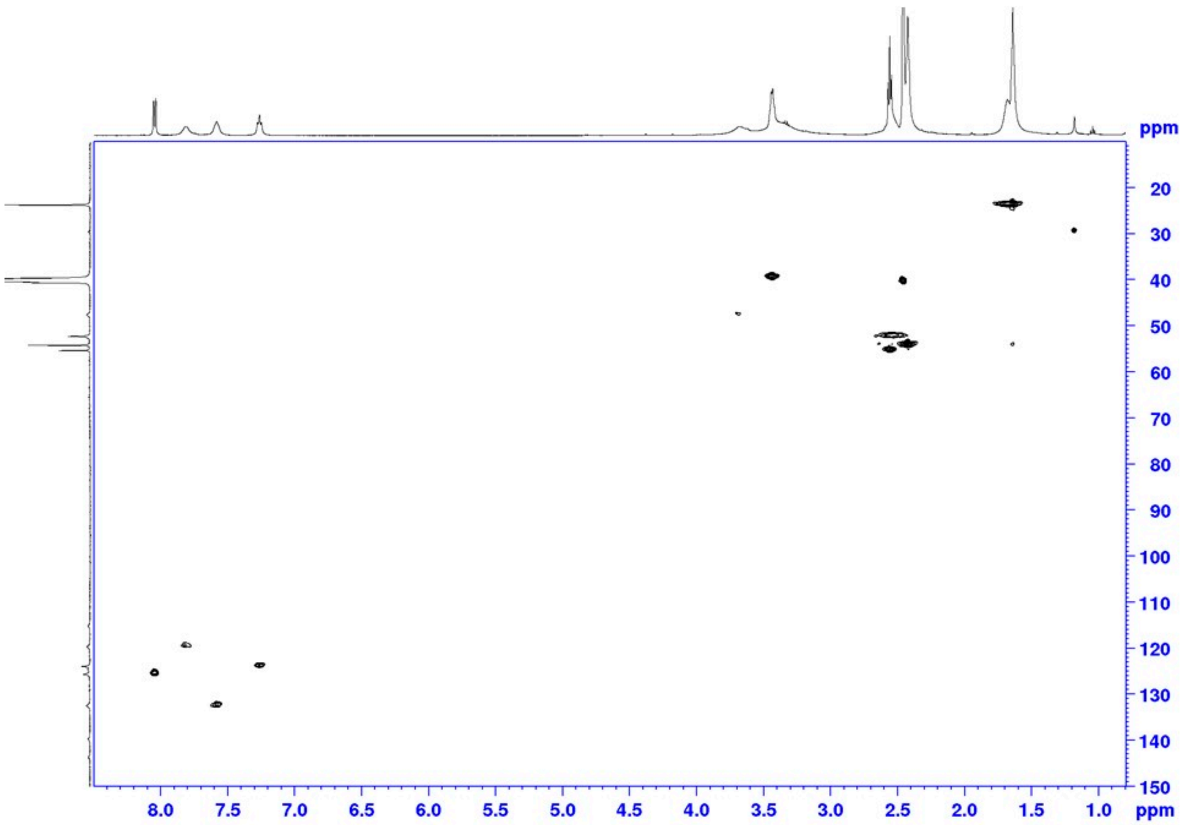

Figure S41. HSQC-NMR spectrum of **19a**

4-oxo-*N*-(2-(pyrrolidin-1-yl)ethyl)-3-(pyrrolidin-1-ylmethyl)-1,4-dihydroquinoline-2-carboxamide (19b)

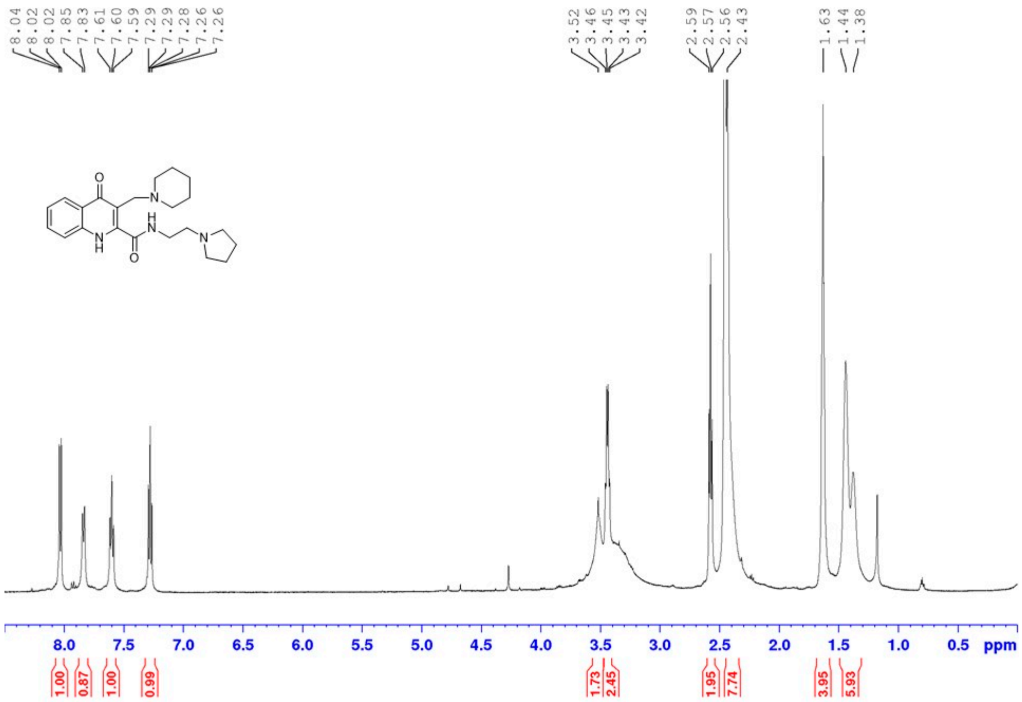

Figure S42. ^{1}H-NMR spectrum of **19b**

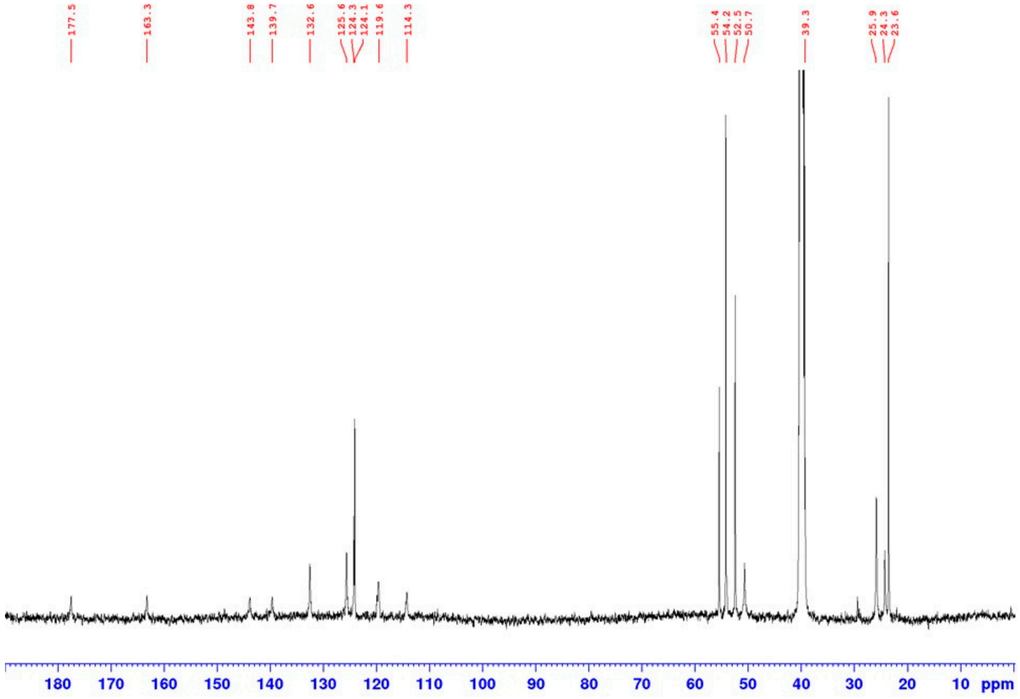

Figure S43. ^{13}C-NMR spectrum of **19b**

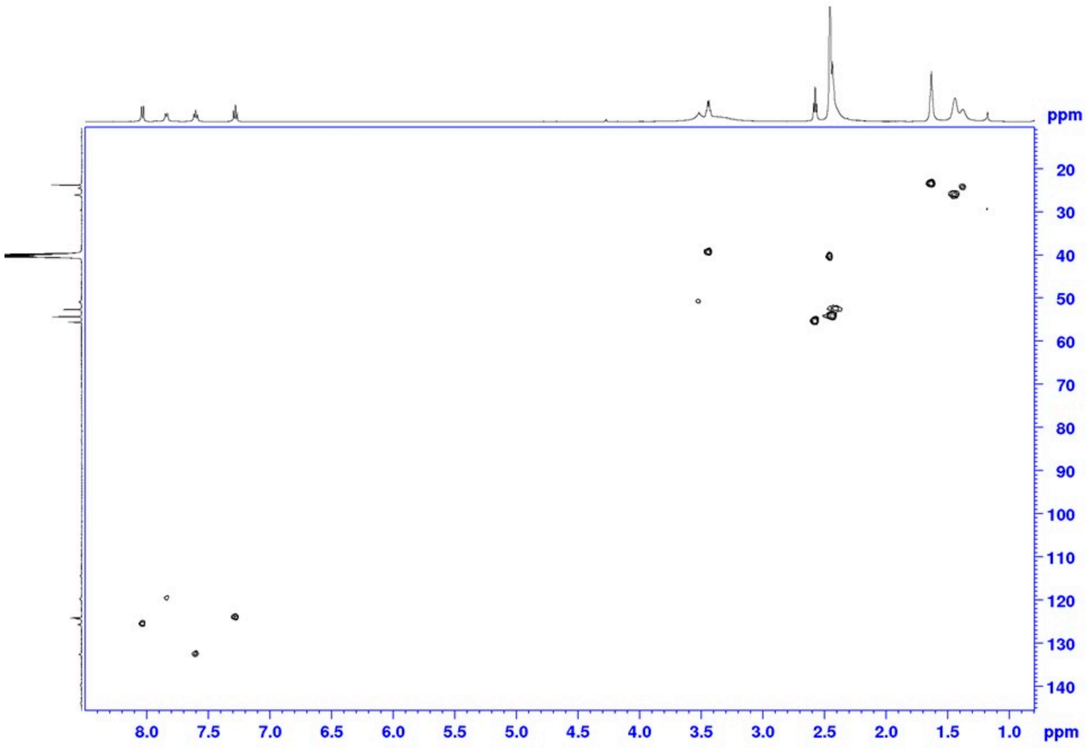

Figure S44. HSQC-NMR spectrum of **19b**

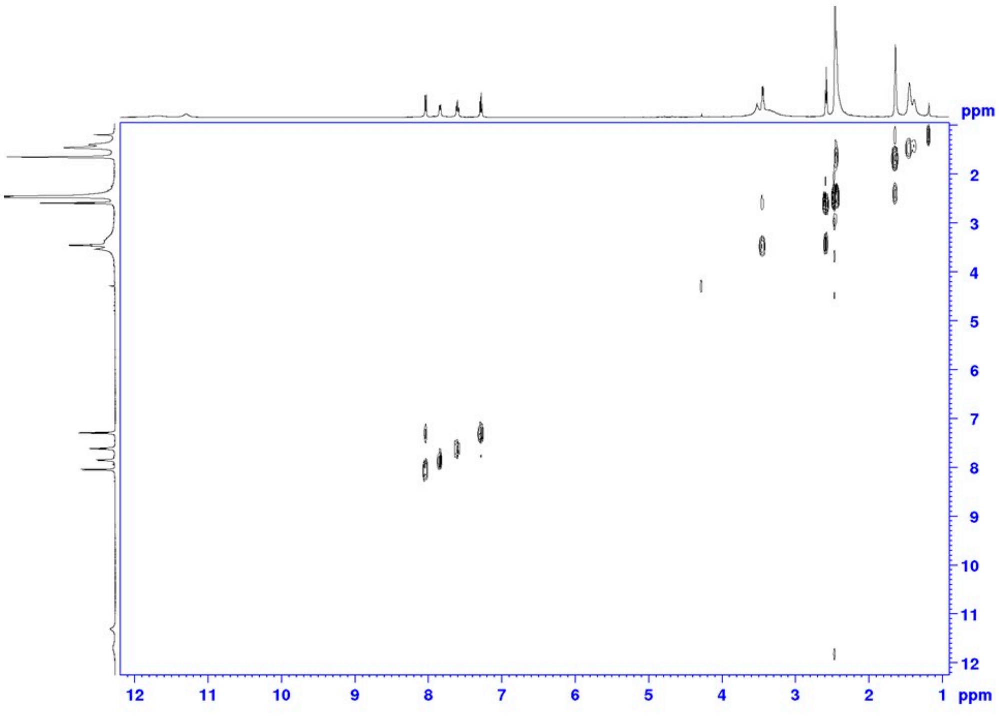

Figure S45. COSY-NMR spectrum of **19b**

4-Oxo-3-(morpholinomethyl)-*N*-(2-(pyrrolidin-1-yl)ethyl)-1,4-dihydroquinoline-2-carboxamide (19c)

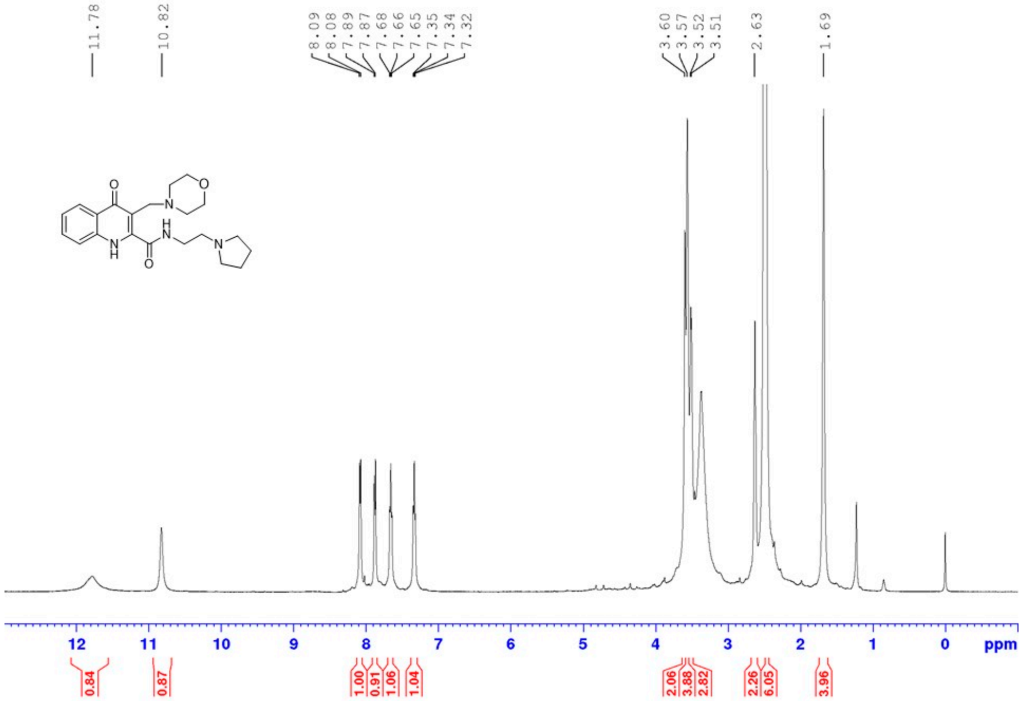

Figure S46. ^{1}H-NMR spectrum of **19c**

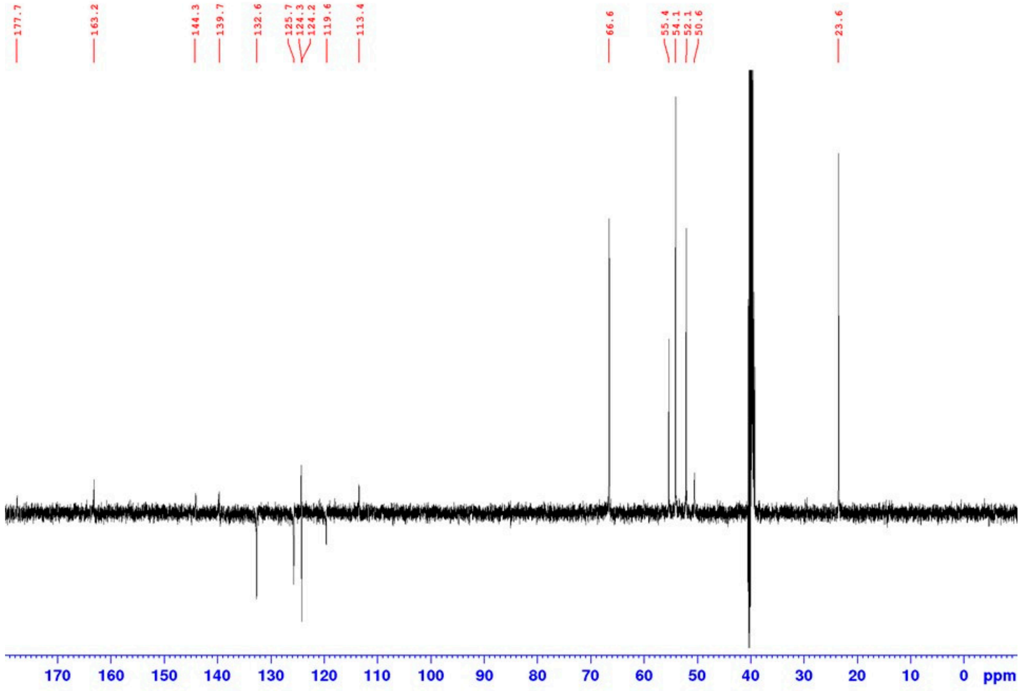

Figure S47. ^{13}C-NMR spectrum of **19c**

4-oxo-1,4-dihydroquinoline-2-carboxamide (20)

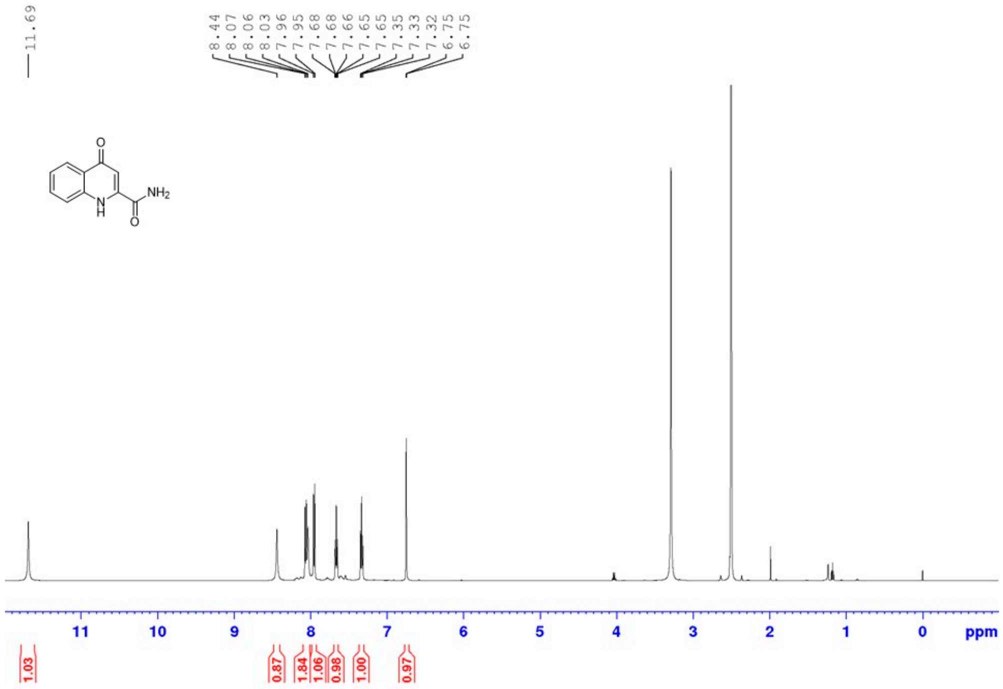

Figure S48. ^{1}H-NMR spectrum of **20**

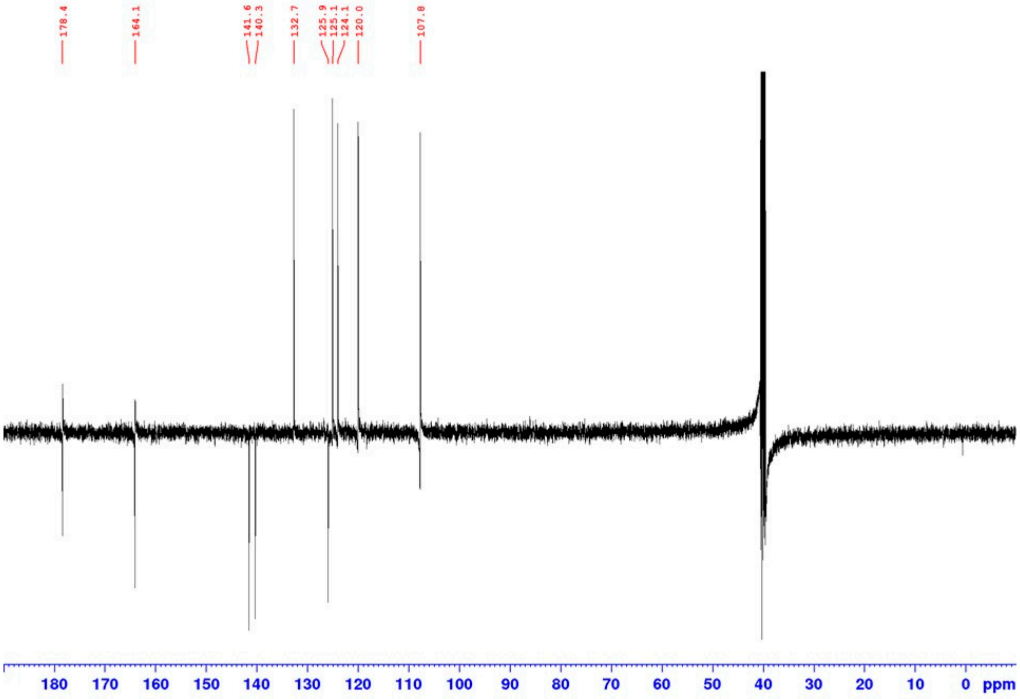

Figure S49. ^{13}C-NMR spectrum of **20**

3-(Morpholinomethyl)-4-oxo-1,4-dihydroquinoline-2-carboxamide (21)

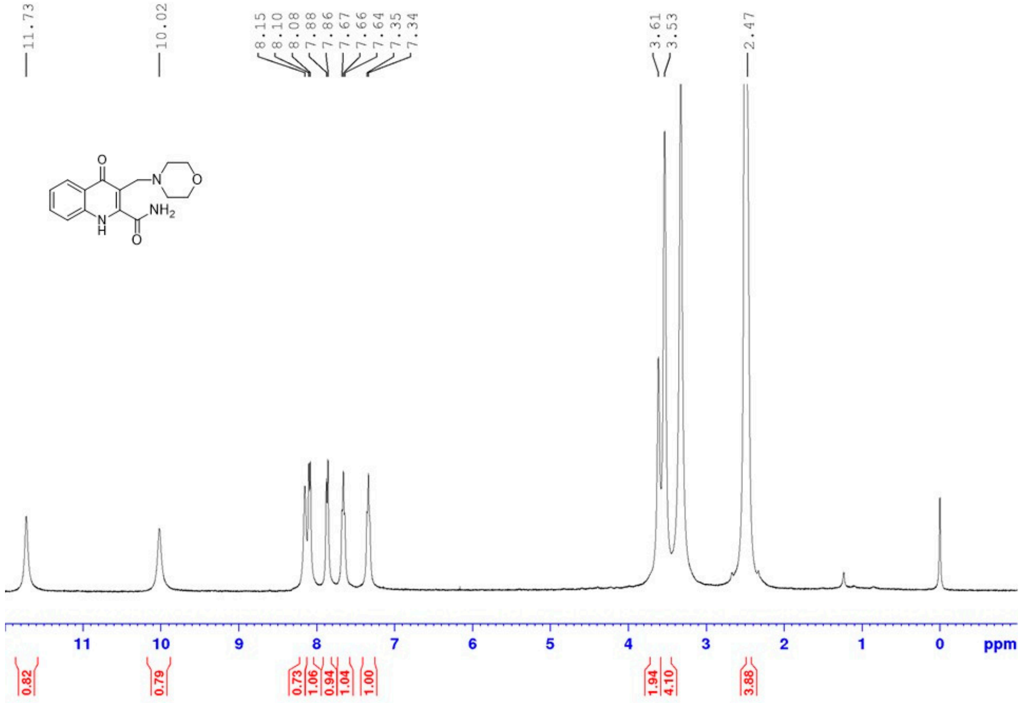

Figure S50. ^{1}H-NMR spectrum of **21**

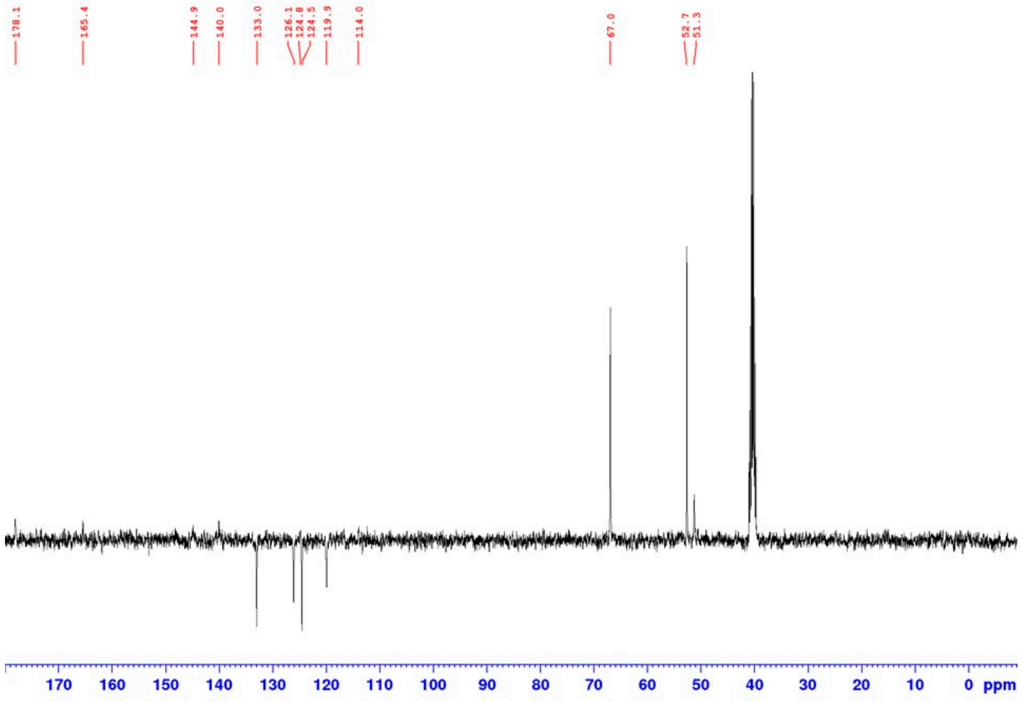

Figure S51. ^{13}C-NMR spectrum of **21**

Methyl 3-(morpholinomethyl)-4-oxo-1,4-dihydroquinoline-2-carboxylate (22)

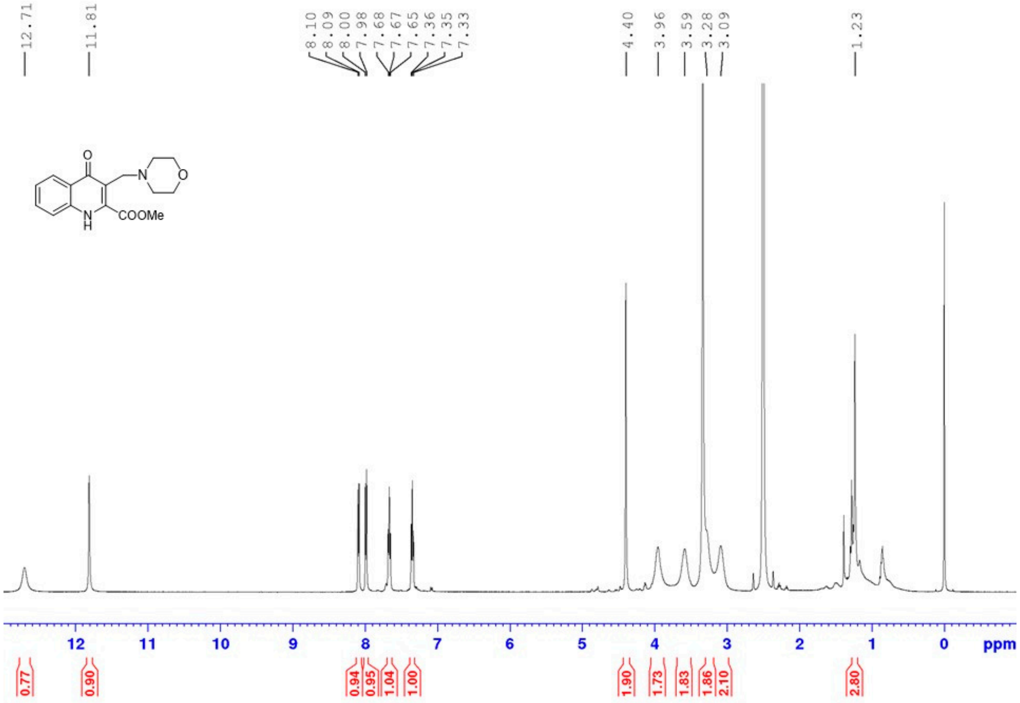

Figure S52. ^{1}H-NMR spectrum of **22**

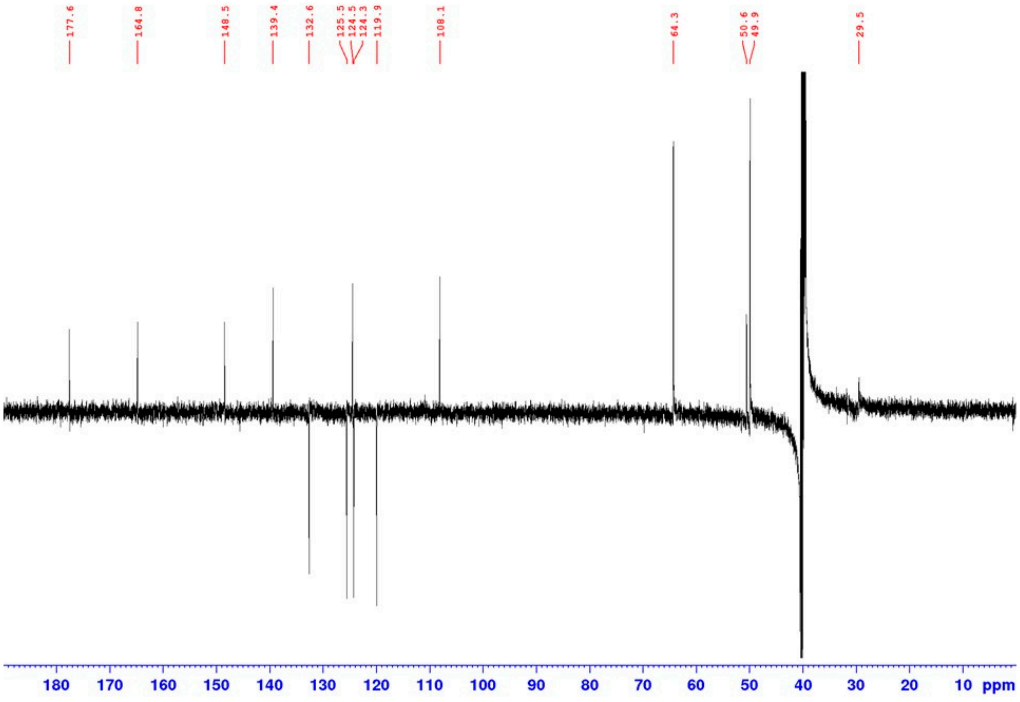

Figure S53. ^{13}C-NMR spectrum of **22**
